# Boreal Rivers as Sources of Terpenoid Emissions

**DOI:** 10.1111/gcb.70540

**Published:** 2025-10-10

**Authors:** Wasi Hashmi, Huizhong Zhang‐Turpeinen, Lukas Kohl, Anne Kuningas, Carlos Palacin‐Lizarbe, Xudan Zhu, Niko Kinnunen, Maija E. Marushchak, Janne Rinne, Anne Ojala, Frank Berninger, Jukka Pumpanen

**Affiliations:** ^1^ Department of Environmental and Biological Sciences University of Eastern Finland Kuopio Finland; ^2^ Department of Environmental and Biological Sciences University of Eastern Finland Joensuu Finland; ^3^ Natural Resources Institute Finland (Luke) Helsinki Finland

**Keywords:** boreal rivers, BVOC, catchment, dissolved organic carbon, monoterpenes, sesquiterpenes

## Abstract

Streams and rivers are biogeochemical hotspots that contribute substantially to inland water CO_2_ emissions. However, their role as sources of biogenic volatile organic compounds (BVOCs) remains poorly understood. We quantified terpenoid emissions from two boreal rivers in peatland and upland mineral soil catchments in Finnish Lapland based on monthly observations over two growing seasons. Both rivers were significant sources of terpenoid emissions, with predicted mean annual emissions of monoterpenes and sesquiterpenes around 4.9 ± 2.8 and 1.2 ± 0.8 mg m^−2^ year^−1^, respectively. The emissions were comparable to those from the boreal forest floor and other ecosystems, and they varied seasonally. The emission composition of the clear water river exhibited unclear seasonal variability and a greater diversity of compounds, while emissions of α‐pinene and p‐cymene showed distinct temporal trends in the brown water river. Dissolved organic carbon concentrations, SUVA_254_, and air temperature were the main drivers of seasonal variation in emissions. Our study revealed that boreal lotic ecosystems are a significant source of terpenoid emissions, which should be considered in the estimations of global ecosystem BVOC emissions.

## Graphical abstract

Boreal rivers are a significant source of natural airborne chemicals called terpenoids, similar to those released by terrestrial ecosystems. We found these rivers emit a variety of terpenoids, with emissions varying by season and river type. Our findings suggest that rivers should be included when calculating global emissions of these compounds.

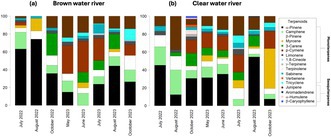

## 1 Introduction

Biogenic volatile organic compounds (BVOCs) are a large group of molecules, varying in size and physicochemical properties, along with metabolic origins (Peñuelas and Staudt 2010; Kesselmeier and Staudt 1999; Laothawornkitkul et al. 2009). Importantly, they affect the radiative forcing of the atmosphere by contributing to cloud formation through the formation of secondary organic aerosols (SOA) and by reducing the oxidation capacity of the atmosphere, prolonging the lifetime of methane (Guenther et al. 2012; Boy et al. 2022). Annual global BVOC emissions from 2001 to 2020 were estimated at approximately 835 Tg year^−1^, with terpenoid emissions comprising 556 Tg year^−1^ (Wang, Wang, et al. 2024) and accounting for 70% of the total global volatile organic compound (VOC) emissions (Guenther et al. 2012, 1995; Wang, Wang, et al. 2024). Vegetation is a major source of diverse BVOCs, with terpenoid emissions (isoprene, monoterpenes, and sesquiterpenes) being the dominant classes. BVOC emissions from vegetation in terrestrial ecosystems have been particularly well characterized (Wang, Wang, et al. 2024; Guenther et al. 1995; Sakulyanontvittaya et al. 2008; Isidorov et al. 1985). Beyond aboveground foliage, BVOCs can also originate from plant roots and the microbial breakdown of plant litter or organic matter in the soil (Tang et al. 2019; Greenberg et al. 2012; Svendsen et al. 2018; Lin et al. 2007).

Marine ecosystems also contribute significantly to global BVOC emissions. Isoprene, dimethyl sulphide, and volatile halocarbons are the major BVOCs released by marine phytoplankton (Zhao et al. 2023). These observations demonstrate that aquatic ecosystems are important for the global BVOC budget. Freshwater ecosystems, such as lakes, rivers, and streams, represent dynamic interfaces between terrestrial and marine environments, playing a critical role in the global carbon (C) cycle and serving as sources of carbon dioxide (CO_2_) and conduits for lateral C transport from land to ocean (Battin et al. 2023). However, current BVOC studies are primarily limited to lacustrine ecosystems and lab observations. The contribution of streams and rivers to BVOC emissions is unknown, and information regarding their sources and underlying mechanisms in lotic systems is limited (Rathbun 2000). Previous studies on other types of VOCs have highlighted the role of rivers and streams as important emitters of pollutant VOCs (Wang, Liu, et al. 2024; Terracciano and O'Brien 1997; Delzer et al. 1996). This gap in knowledge hampers a complete understanding of the impacts of BVOC emissions on the climate. The importance of exploring BVOC emissions from aquatic subarctic and boreal ecosystems arises from arctic rivers draining ~15% of the global land surface (Feng et al. 2021), having a drainage area of 22.1 million km^2^.

The importance of lateral C transport from terrestrial to aquatic ecosystems has already been recognized. The proportion of lateral dissolved organic carbon (DOC) flux is estimated to be significant of the total C budget (10%–30% of catchment net ecosystem exchange, NEE), especially in peatland‐dominated catchments (Olefeldt et al. 2013). On a global scale, the lateral transport of C from terrestrial ecosystems to inland waters is ca. 2.95 ± 0.54 Pg C year^−1^ (Regnier et al. 2022). Part of this C is processed in aquatic ecosystems, ending up as greenhouse gases (GHGs) and other gases; part is buried in sediments; and finally, approximately 0.95 ± 0.25 Pg ends up through estuaries into the oceans (Regnier et al. 2022). Despite covering only a small proportion of the Earth's surface, rivers act as “biogeochemical reactors” that metabolize organic C (OC) to GHGs (Battin et al. 2023). This highlights their potential importance also in the fluxes of other climate‐relevant gases, such as BVOCs. We believe that due to low aquatic biomass in the boreal rivers (Schindler 1998), aquatic degradation of DOC by photochemical and microbial processes can be a source of BVOCs. Sunlight breaks down DOC into smaller, volatile molecules, while microbes consume DOC and release BVOCs as a metabolic byproduct. Recent research has identified a new process: the heterogeneous oxidation of dissolved organic matter (DOM) at the sea surface produces carbonyl‐containing VOCs. However, the overall importance of this process is not yet understood (Zhou et al. 2014; Ciuraru et al. 2015).

The partitioning of terpenoids between the atmosphere and water is largely governed by Henry's law volatility constants (*H*_v_), which reflect the balance between volatility and solubility and drive their emission rates from aquatic ecosystems. Terpenes are poorly soluble in water and highly volatile (Kesselmeier and Staudt 1999; Fichan et al. 1998; Martins et al. 2017; Weidenhamer et al. 1993), while sesquiterpenes have much lower volatility and solubilities compared to monoterpenes due to their larger size (Copolovici and Niinemets 2015; Hui et al. 2023). Volatilization of terpenes is also strongly temperature‐dependent; the *H*_v_ of monoterpenes increases by 1.3–1.8‐fold for every 10°C rise in temperature (Copolovici and Niinemets 2005), while that of sesquiterpenes increases by about 1.5‐fold for a 10°C increase (Copolovici and Niinemets 2015). The transport, fate, and behavior of terpenoid emissions from aquatic ecosystems are governed by a complex interplay of physical, chemical, and biological processes (Rathbun 2000). Seasonality, changes in environmental factors, such as microclimatic conditions of the catchment soil, vegetation patterns, and associated abiotic factors like temperature, humidity, and photosynthetically active radiation (PAR) determine local BVOC emissions in terrestrial ecosystems (Aaltonen et al. 2011). However, so far, BVOC emissions and their seasonal variation in lotic systems in boreal ecosystems have not been quantified or characterized, leaving a critical gap in understanding their role in climate feedback mechanisms.

Arctic and boreal regions are undergoing rapid environmental changes due to climate warming, which alters hydrological processes, DOC export, microbial activity in aquatic systems, and temperature‐sensitive BVOC fluxes (Wrona et al. 2016; Baggesen et al. 2021; Faubert et al. 2010; Lindwall et al. 2016). Differences between catchment types are another defining feature that can further influence BVOC emissions because of the differences in the quantity and quality of DOC input from the catchment area. Therefore, the connection of aquatic systems to the surrounding catchment area should be considered due to their strong influence on water quality. Peatland‐based catchments are often characterized by large amounts of organic matter, which makes them capable of storing large water quantities, but despite this, they are poor suppliers of baseflow to streams and rivers (Holden and Burt 2003). This is because peatlands are usually diplotelmic, which means they have an upper peat layer consisting of roots and recently decomposing plant material above a more decomposed peat layer that is denser, more decomposed, and has poor hydraulic conductivity (Ivanov 1981). By contrast, the water in upland mineral soil rivers is usually discharged from groundwater sources with smaller variation in seasonal flow conditions. Furthermore, alkaline soil tends to retain more BVOCs than acidic soils (Tang et al. 2019), and both organic matter and pH together affect VOC emissions (Abis et al. 2018). These contrasting catchment types provide a natural framework for exploring how BVOC emissions in rivers are influenced by lateral C transport, soil type, and hydrological dynamics.

Addressing these knowledge gaps is vital because BVOC emissions from lotic systems could contribute to regional atmospheric chemistry and climate impacts, particularly in high‐latitude ecosystems that are highly sensitive to environmental change. Here, we report for the first time the terpenoid emissions from boreal rivers measured in two catchments dominated by contrasting soil types (peat and mineral soil) in the high‐latitude boreal region. The river from the peatland catchment is hereafter referred to as the brown water river (BW), while the river from the mineral catchment will be called the clear water river (CW). We quantify and identify the varying BVOC compounds, focusing on terpenoids, mainly C_*n*5_H_*n*8_ (monoterpenes (MT) and sesquiterpenes (SQT), whereas isoprene was always below the detection limit), that were released from these two catchments over a span of 2 years (2022–2023). We also investigated the impact of seasonal variation in river flow rate, DOC concentration, and other environmental variables on the emissions. Furthermore, we predicted both monthly and annual MT and SQT emissions using multiple linear regression modeling to compare the scale of river emissions.

We address the following research questions: (i) how much MT and SQT are emitted by the BW and CW rivers, (ii) what are the chemical compositions of these emissions, (iii) which environmental and hydrological parameters drive the emissions, and (iv) how do emission rates from rivers compare with other boreal ecosystems.

## 2 Materials and Methods

### 2.1 Study Sites

**FIGURE 1:**
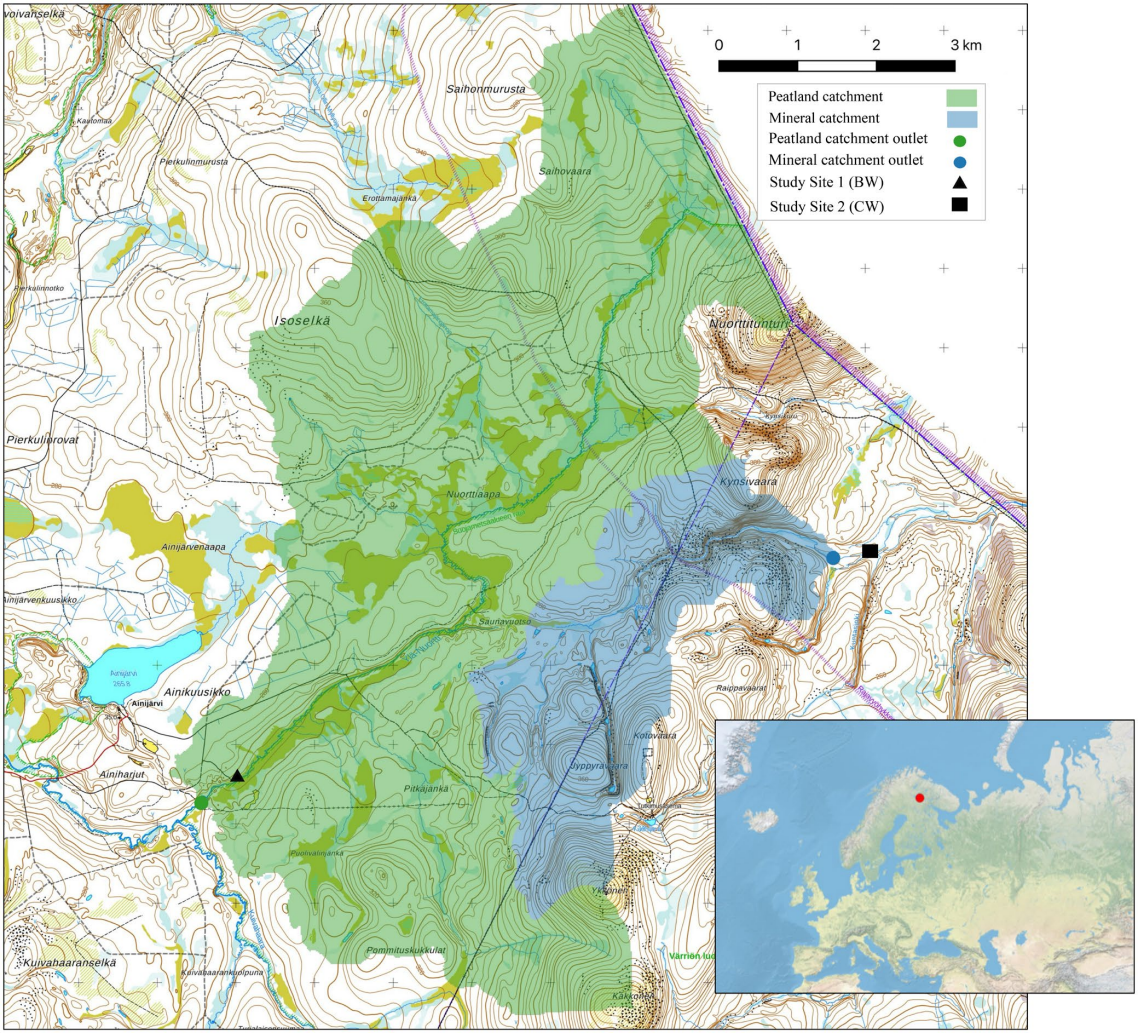
Map of study site showing the extent of peat (green area) and mineral (blue area) catchment coverages, site 1 is a brown water river (Yli‐Nuortti River) and site 2 is a clear water river (Kotkakurunoja River), map credit to Jari‐Pekka Nousu. Map lines delineate study areas and do not necessarily depict accepted national boundaries.

The study areas were a BW river, Yli‐Nuortti (67°46′8″ N, 29°12′43″ E), located on a peatland‐dominated catchment, and a CW river, Kotkakurunoja (67°78′44″ N, 29°68′93″ E), located on an upland mineral soil catchment (Figure 1). Yli‐Nuortti River catchment has an approximate peatland coverage of 20%, consisting mainly of open aapa mires with minerotrophic fen vegetation (Saarela et al. 2024); Kotkakurunoja River flows through an upland mineral soil catchment with a peatland coverage of < 1%. Catchment area soil mainly consists of glacial till (Saarela et al. 2024). Based on visual observation, the rivers had different dominating aquatic submerged macrophytes. Mare's‐tail ( *Hippuris vulgaris* ) was the most abundant aquatic plant growing in the BW river, while brook moss (*Fontinalis* sp.) (Figure S1) was abundant in the CW river. However, we did not carry out biomass inventory in the rivers. The measurement campaign was spread out between July 2022 and October 2023 and was carried out a total of eight times. Both rivers are accessible via the Värriö subarctic research station, managed by the University of Helsinki, Finland. The Värriö region is defined by its subarctic climate characteristics, has no underlying permafrost, and the topsoil in the region is mainly haplic podzol on glacial till (Saarela et al. 2024). Peatlands in the area are mainly aapa mires or forested peatlands. The average annual precipitation is 592 mm, the average yearly air temperature is −1.0°C, and snow cover duration ranges between 200 and 225 days/year. The growing season is restricted to 105–120 days (Saarela et al. 2024). Meteorological conditions consisting of continuous PAR, air humidity, precipitation, and air temperature are shown in Figure S4 and were acquired from the continuous measurement database at the SMEAR research station (https://smear.avaa.csc.fi/) run by the University of Helsinki.

### 2.2 BVOC Measurement From Sampling Sites

Quantification of BVOC emissions was conducted using the dynamic headspace chamber technique. During each individual sampling campaign, four chamber measurements were taken from each site. A total of 64 samples and 32 blanks were collected over the entire sampling period 2022–2023. BVOC collection was performed with the aid of sampling chambers that consisted of an aluminum collar (diameter = 22 cm) and a chamber frame (height = 27 cm). To form a dome‐shaped chamber, a transparent polyethylene terephthalate bag (PET bag), with dimensions of 45 × 55 cm and providing a total volume of 6 L, was inserted over the chamber frame. The bags were preconditioned and sterilized prior to use by placing them in the oven at 120°C for 1 h. We used chambers that were always placed in the same position in the middle of each river. This approach was chosen for two key reasons:

1. It ensured consistent sampling conditions across different seasons and flow rates, allowing for a direct comparison between the two rivers. This was crucial for isolating the effects of water quality and catchment type on BVOC emissions and avoiding the potential differences between the rivers resulting from different water flow rates and turbulence.
2. It was a logistical necessity, as the long measurement times would have caused the chambers to drift to the riverbanks, and the challenging terrain made a large‐scale spatial sampling design impossible.

Furthermore, these fixed locations were equipped with long‐term monitoring stations for DOC concentration, water temperature, and flow rate (e.g., Zhu et al. 2021, 2022), providing valuable background data. The measurements were made throughout the day, from morning until evening. The chambers were flushed with filtered air with the help of carbon filters and were scrubbed with ozone at a flow rate of 230 mL min^−1^ for 30 min. Adsorbent tubes (Markes International Ltd., Llantrisant, UK) were attached to the headspace of the chamber. Air samples were collected by sucking air through the adsorbent tube at a 210 mL min^−1^ flow rate for 120 min. For the mixing of chamber air during both the flushing and sampling processes, an electric fan was used inside the chamber (diameter = 5 cm). A total of four blanks per measurement were conducted by placing the chamber inside the PET bags, with the air being flushed for 20 min, followed by the collection of air samples from the headspace of the chamber into adsorbent tubes for a period of 30 min.

During these measurements, the air temperature and relative headspace humidity were measured via a thermohygrometer (Testo 605‐H1‐Mini Thermohygrometer, Testo Ltd., Alton Hampshire, UK). The water temperature in the river bodies was monitored through a portable probe (P 300w, Dostmann Electronic GmbH, Germany). An oxygen sensor (Fibox 4 Trace, Oxygen Meter, Precision Sensing, Germany) was utilized for measuring the oxygen concentrations of the river water in the designated sampling sites. PAR above the chamber was measured using the Delta‐T Quantum sensor (Delta‐T Devices Limited, Cambridge, UK).

### 2.3 CO_2_ Flux Measurements

The CO_2_ fluxes from the two streams were measured using the manual floating chamber technique. Four replicate chamber measurements were performed at both sites (Bastviken et al. 2015). The floating chambers were made of polypropylene plastic buckets (Orthex, Espoo, Finland), with floats and sampling outlets made of cellular plastic (Gålfalk et al. 2013). The lower edge of each chamber was submerged 3 cm under water, and the height of the chamber above the water surface was 13 cm. The surface area of each chamber was 0.12 m^2^, and the headspace volume was 0.009 m^3^. Air sampling from the chamber headspace was conducted at 0, 3, 7, 14, and 21‐min intervals using a 60‐mL BD PlastipakTM syringe connected to a BD Connecta 3‐way stopcock valve (Becton, Dickinson and Company, NJ, USA). A 24‐mL air sample was injected into 12‐mL Labco Exetainer vials (Labco Ltd., Lampeter, Ceredigion, UK) that were flushed with nitrogen (N_2_) and pre‐evacuated prior to use. An Agilent 7890B gas chromatograph (Agilent Technologies, Palo Alto, CA, USA) equipped with a Gilson liquid handler GX271 autosampler (Gilson Inc., Middleton, WI, USA) was used to analyze the collected gas samples for CO_2_ concentrations.

### 2.4 DOC and Total Nitrogen (TN) Measurements and Optical Analyses

Water samples were collected from the water surfaces of the river up to a 10‐cm depth near our BVOC measurement spot in 50‐mL Falcon tubes (Thermo Scientific, MA, USA) and stored at −18°C until further analysis. A standardized method (SFS‐EN 1484) using the total organic carbon (TOC) analyzer (Shimadzu TOC‐VCPH, Shimadzu Corp., Kyoto, Japan) was followed to determine the DOC and TN concentrations in the collected samples. Absorbance measurements from the water samples were conducted at 254 nm using a 1‐cm quartz cuvette Shimadzu UV‐2401 (Shimadzu Co., Kyoto, Japan) and a laboratory benchtop spectrophotometer (UV1800, Shimadzu, Kyoto, Japan). SUVA_254_ was calculated by dividing the absorbance at 254 nm by the DOC concentration. The DOC concentration and turbidity values in both rivers were also continuously monitored in situ using real‐time spectral absorbance in situ portable multiparameter UV–Vis probes (spectro::lyzer, s::can Messtechnik GmbH, Austria) installed in both rivers (Zhu et al. 2022).

Every month, water samples were collected twice a week from both rivers, and they were analyzed for DOC concentration, pH, EC, and SUVA_254_ in the lab. Later, the continuous DOC measurements were calibrated using lab DOC data from laboratory measurements.

### 2.5 Water Chemistry Analysis

Concentrations of chloride (Cl^−^), sulphate (SO_4_^−2^), phosphate (PO_3_^−^), nitrite (NO_2_^−^), and nitrate (NO_3_^−^) were measured using ion chromatography (Dionex ICS‐2100, Thermo Scientific). Prior to the analysis, filtration was conducted by passing the samples through a membrane filter with a pore size of 0.45 μm (Minisart (R) cellulose acetate filters NML, Sartorius). A pH meter (WTW, pH 340) and an EC meter (WTW pH/cond 340i) were subsequently used to measure the pH and electrical conductivity (EC) of the water samples.

### 2.6 Water Flow Rate Measurements

Throughout the sampling campaign, water depth was constantly monitored in 30‐min intervals using pressure sensors that measured the hydrostatic pressure (Levelogger, Solinst, Georgetown, Canada). These sensors were located on the riverbed, with the hydrostatic pressure measurements being corrected by barometric pressure measurements (Barologger, Solinst, Georgetown, Canada). The measurements of the rivers' flow rates were carried out using a manual flow rate meter (Schiltnecht MiniAir2, Schiltnecht Messtechnik, Switzerland). The continuous flow rates of each river were calculated by regression analysis with water depth and manual flow rate measurements during the BVOC measurements.

### 2.7 Laboratory Analysis and Data Processing for BVOC Emissions

Samples collected in the Tenax TA Tubes were analyzed for the quantification and characterization of BVOCs using a scan mode Thermodesorption instrument (ATD400; Perkin Elmer, Wellesley, MA, USA). This was connected to a gas chromatography‐mass spectrometer (GC–MS) (Hewlett‐Packard 6890, MSD 5973; Hewlett‐Packard, Palo Alto, CA, USA). Thermal desorption of the sample took place at a temperature of 325°C for a total timespan of 3 min. The sample was then cryofocused at a temperature of 30°C. In an HP‐5 capillary column (film thickness = 60 m × 0.25 mm, 0.25 μm), the compounds were divided with the aid of helium (He) acting as a carrier gas. During the analysis, the oven temperature was initially kept at 40°C for 6 min and then gradually increased to 125°C at a rate of 5°C min^−1^, with the final temperature increased to 260°C at a steady rate of 10°C min^−1^.

Following this, the TA tubes were then injected with commercial standard mixtures from pure compounds in methanol, using a calibration solution loading rig (Markes International Ltd., Llantrisant, UK) using N_2_ as a carrier gas. We identified the most abundant BVOCs and those belonging to MTs and SQTs. Isoprene was present, but we were unable to quantify it, as the peaks were under the detection limit.

Those detected compounds that were excluded from the standard compound mixtures were recognized via the mass spectra in the Wiley and NIST data library and were later quantified on similar chemical compound structures (α‐pinene for MTs). The chromatograms were identified using the Enhanced ChemStation software (G1701EA Revision E.02.01 on 19 April 2017; Agilent Technologies, Santa Clara, CA, USA). For this, the following equations were used for calculating BVOC concentrations and emissions, respectively (Zhang‐Turpeinen et al. 2020):

(1) $$\begin{array}{c} \text{BVOC concentration}\;\left[\mathrm{\mu g}\;L^{-1}\right]=\\ \frac{\left(\text{BVOC mass in the}\;\mathrm{ATD}\;\text{tube}\;\left[\mathrm{\mu g}\right]-\text{average BVOC mass in the}\;\mathrm{ATD}\;\text{tubes used for blank measurements}\;\left[\mathrm{\mu g}\right]\right)}{\left(\text{Sampling time of}\;120\;\left[\min\right]\times \mathrm{Air}\;\text{flow through}\;\mathrm{ATD}\;\text{tube}\;0.2\;\left[L\;\min^{-1}\right]\right)} \end{array}$$

where ATD represents the Adsorbent Tenax TA tube used.

(2) $$\begin{array}{c} \text{BVOC emission}\;\left[\mathrm{\mu g}\;\text{BVOC}\;m^{-2}\;\text{ground area}\;h^{-1}\right]=\\ \frac{(\text{BVOC concentration}\;\left[\mathrm{\mu g}\;L^{-1}\right]\times \left(\mathrm{Air}\;\text{flow pumped into chamber}\;\left[L\;\min^{-1}\right]\times 60\;\min\right)}{\left(\text{Surface area of collar}\;\left[m^{2}\right]\right)} \end{array}$$

### 2.8 Statistical Analysis

We did not attempt to distinguish differences between rivers in general but restricted our analysis to identifying statistical differences between the two specific rivers in our study. We therefore considered the individual chamber measurements carried out (*n* = 4) during each sampling campaign as independent replicates. Differences between the two rivers were assessed using a Mixed Effects Model (MEM) with river specified as a fixed effect and sampling event as a random effect. To evaluate interannual differences, a separate model was employed that included river and year as fixed effects and month as a random effect. To test for differences between rivers at individual sampling events, a *t*‐test was performed. To identify differences between sampling events, a two‐way ANOVA was conducted with river and sampling events as independent factors. Post hoc comparisons were then made using a Tukey HSD test (Table S4). Prior to statistical tests, the available variables underwent normality assessment using the Shapiro–Wilk test. Log transformations were conducted when the variables violated assumptions of normality, and the Kruskal–Wallis test with a post hoc Dunn test was used in such cases. Furthermore, the standard deviation (SD) of the measured emission values was calculated. In addition, the differences in the BVOC emission profiles between the rivers and various sampling times and their relationships with environmental variables (CO_2_ fluxes from the river, PAR, air temperature, water temperature, pH, EC, DOC, SUVA_254_, TN, Cl^−^, SO_4_^−2^, PO_3_^−^, NO_2_^−^, and NO_3_^−^) were characterized using RDA analysis. Data for RDA were scaled and centralized and included BVOC compounds and environmental variables. The graphical representation and statistical analysis for this research were created on the R version (RStudio Team, 2024, with RStudio utilizing version 12.1, Posit Software, PBC).

### 2.9 Linear Regression Model and Predictions

The factors affecting the variation in BVOC emissions were studied using a multiple linear regression (MLR) model, where the BVOC emissions in both rivers were used as dependent variables (Tables S2 and S3 present the model structure in tables). Air temperature, water flow rate, river water DOC concentration, EC, PAR, and SUVA_254_ were used as independent variables. Model fitting was performed using stepwise removal. The Akaike information criterion (AIC), generalized variance inflation factor (GVIF), and the adjusted *R* ^2^ were calculated for each model, and the models with the lowest AIC were regarded as the best models. We also considered *p*‐values, GVIF, and adjusted *R* ^2^ when the models were simplified. Multicollinearity was addressed during model refinement, ensuring reliable results. The best models, i.e., model 10 for MT emissions and model 9 for SQT emissions (Table 1), incorporated all significant components, which ensure no overfitting issues. The MLR was carried out using the R package “*lm*.” The predictions of MT and SQT emissions during the open‐water period were calculated using MLR models 10 and 9, respectively, and continuous data from DOC concentration and air temperature.

**TABLE 1:** Simplified MLR model summary, the first three models (models 8, 9, and 10) show monoterpene (MT) and the last three (models 7, 8, and 9) models show sesquiterpene (SQT) models.

Model		*F*‐statistic	*p*‐value	Multiple *R* ^2^	Adjusted *R* ^2^	AIC	Variables	GVIF	Estimate	Std. error	*p*‐value
8	MT emissions~Rivers + Flow rate + DOC	7.2	0.01	0.64	0.55	7.52	(Intercept)	—	−1.90	0.4	0.0009***
							Rivers	1.6	0.55	0.2	0.01**
							Flow rate	2.1	−3.60	2.5	0.18
							DOC	2.2	2.21	0.5	0.001***
9	MT emissions~Rivers + Air temperature + DOC	7.1	0.01	0.64	0.55	7.64	(Intercept)	—	−1.78	0.4	0.002**
							Rivers	1.7	0.54	0.2	0.007**
							Air temperature	1.2	0.01	0.0	0.19
							DOC	1.8	1.53	0.5	0.008**
10	MT emissions~Rivers + DOC	9.0	0.003	0.58	0.52	7.99	(Intercept)	—	−1.86	0.5	0.001**
							Rivers	1.5	0.61	0.2	0.003**
							DOC	1.5	1.79	0.5	0.002**
7	SQT emissions~Air temperature + Suva_254_ + EC	8.0	0.003	0.67	0.58	−16.0	(Intercept)	—	−0.41	0.19	0.05
							Air temperature	1.2	0.01	0.00	0.07
							Suva_254_	1.1	−0.19	0.06	0.01*
							EC	1.1	0.00	0.00	0.15
8	SQT emissions~Air temperature + Suva_254_	9.8	0.003	0.60	0.54	−15.1	(Intercept)	—	−0.33	0.19	0.11
							Air temperature	1.1	0.01	0.005	0.03*
							Suva_254_	1.1	−0.17	0.07	0.02*
9	SQT emissions~Air temperature	8.8	0.010	0.39	0.34	−10.2	(Intercept)	—	−0.80	0.08	0.00
							Air temperature	—	0.02	0.01	0.01*

*Note:* For each model, the table presents overall goodness‐of‐fit metrics (overall *F*‐statistic and *p*‐value, multiple *R*‐squared [*R* ^2^], adjusted *R*‐squared [adjusted *R* ^2^], and Akaike information criterion [AIC]), along with details for each predictor variable: Coefficient estimate, standard error, *p*‐value, and generalized variance inflation factor (GVIF). Significant codes for statistical test outputs: ***p < 0.001, **p < 0.01, *p < 0.05.

Further, we examined the relationship between terpenoid emissions and two environmental factors: flow rate and wind speed using linear regression. The analysis was conducted separately for each site (BW and CW). The R script for models and figures is available at https://doi.org/10.6084/m9.figshare.28919453).

## 3 Results

### 3.1 Emission Patterns

**FIGURE 2:**
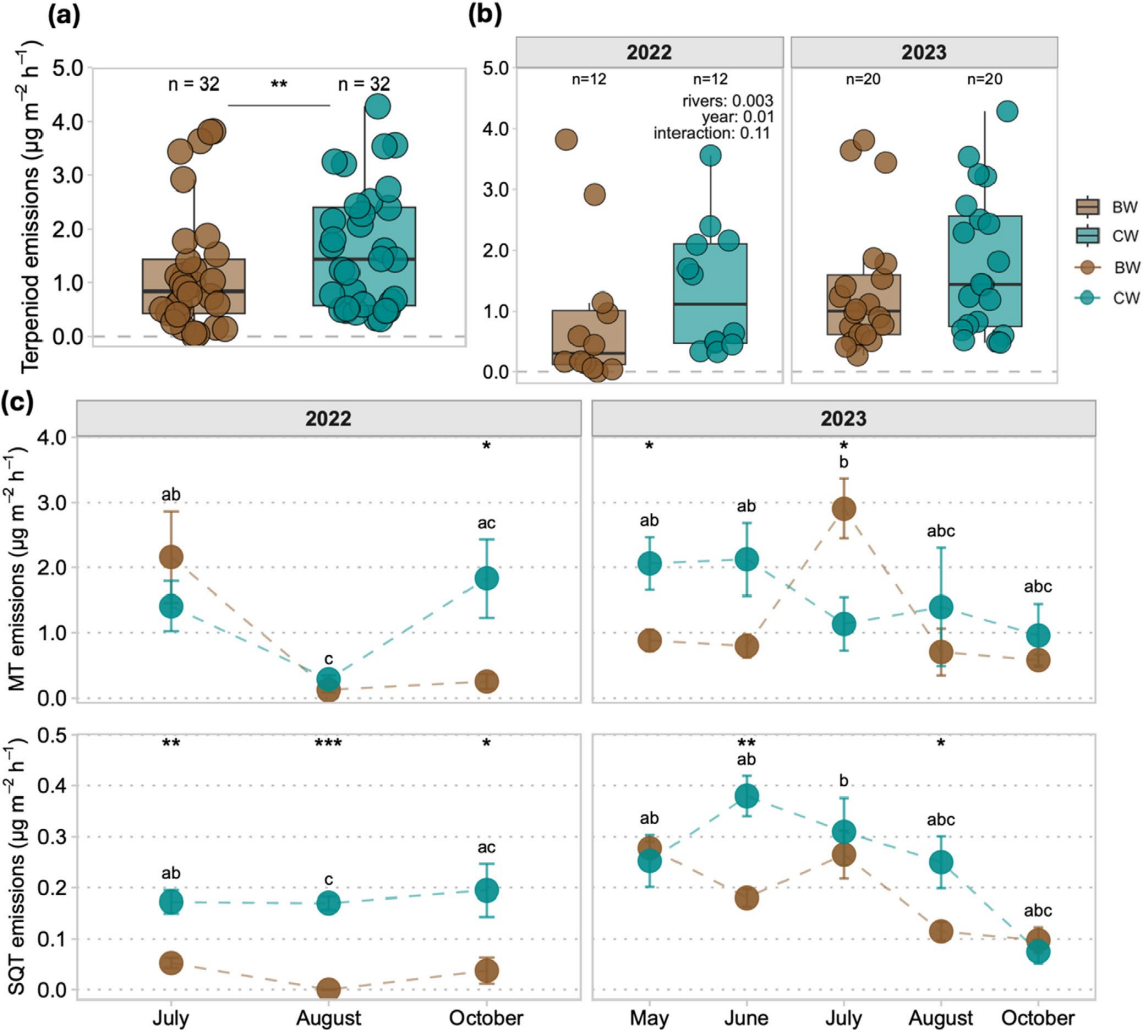
Overall emission profile from two rivers, (a) Terpenoid emissions in the brown water and clear water river and (b) in different years. Box plot showing lower and upper quartiles, median (thick black line), smallest and largest values with outliers (thin line), and circle in (a) and (b) represents individual observation from the studied river. Mixed effects model (MEM) for difference between the emissions of the two rivers and years. (c) Average monoterpene (MT) and sesquiterpene (SQT) emissions in the measurement campaigns with SD shown as error bars; *n* = 4 dots in (c) represent the average of replicates at each studied river during a single campaign. *T*‐test for differences between rivers during individual sampling events and Two‐way ANOVA with rivers and sampling event as independent factors followed by a Tukey HSD post hoc test (shown by letters). Significant codes for statistical test outputs for *t*‐test, mixed effect model shown by: ***p < 0.001, **p < 0.01, *p < 0.05.

Both rivers acted as sources of terpenoids to the atmosphere. The overall average terpenoid emissions were (mean ± SD) 1.2 ± 1.1 and 1.6 ± 1.1 μg m^−2^ h^−1^ in the BW and CW rivers, respectively. The emissions in the CW river were higher, leading to a significant statistical difference between the emissions of the two rivers (mixed effects model (MEM) *p*‐value = 0.008) (Figure 2a). There were also differences in the emissions between both studied years (*p*‐value, rivers = 0.003, year = 0.01 and interaction = 0.11) (Figure 2b). Seasonally, the emissions from BW and CW rivers showed distinct differences between months (*t*‐test shown as asterisks in Figure 2c, *p*‐value ranging from = 0.02–0.001). MTs were the most abundant VOC group in the emissions in both rivers. The MT emissions from the BW river exhibited distinct maxima during July in both years (2.2 ± 1.4 and 2.9 ± 0.9 μg m^−2^ h^−1^, respectively), being two times higher than emissions from the CW river, whereas the MT emissions from the CW river were highest during shoulder seasons (October 2022 and May–June 2023), when they were nearly twice as high as those in the BW river during autumn and earlier summer, respectively (Figure 2c). Throughout the whole measurement period, SQT emissions from the CW river were usually higher (except May and October 2023) compared with the BW river (Figure 2c).

### 3.2 Emissions Composition

**FIGURE 3:**
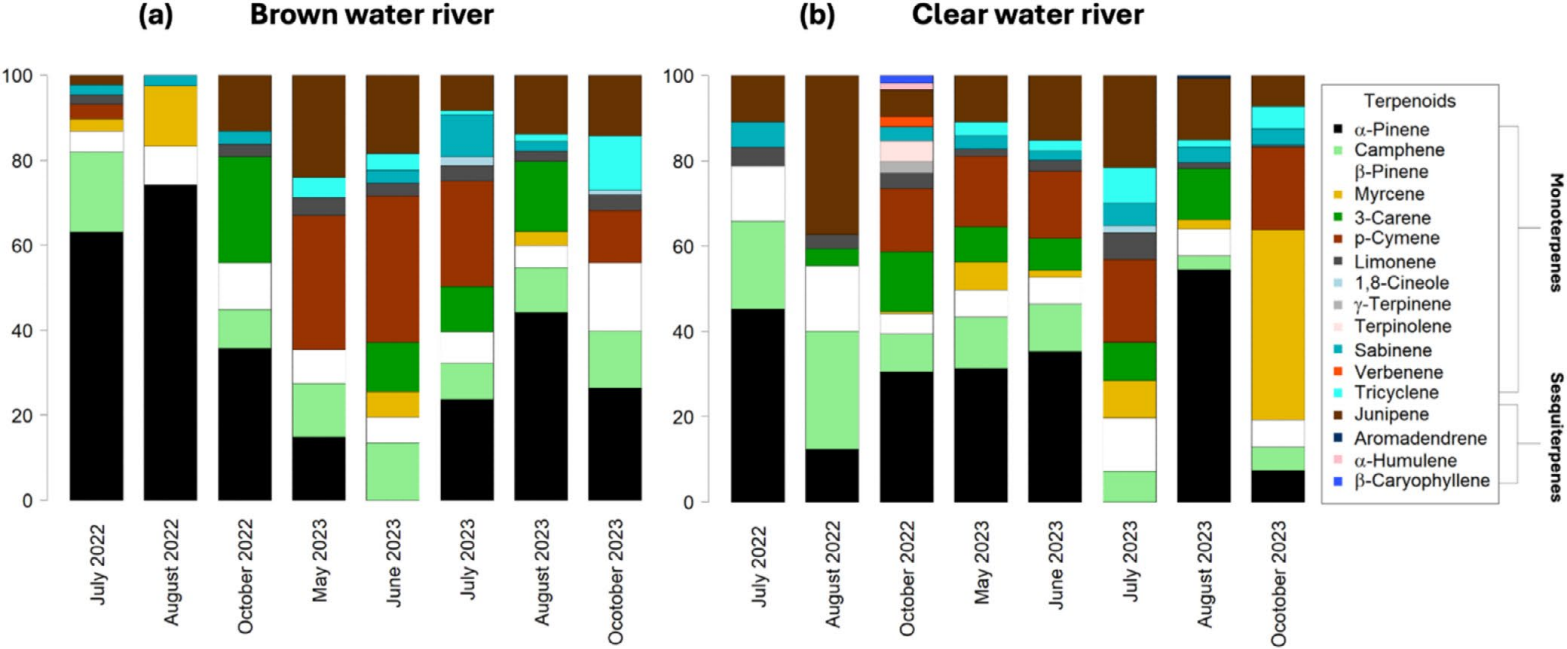
Percentage composition of terpenoid emission profiles in (a) brown water river and (b) clear water river from 2022 and 2023.

MT emissions accounted for 80% of the total terpenoid emissions. Only a few SQTs (20%) were present in the emission profile, with junipene being the most abundant SQT compound (Figure 3). Overall, α‐pinene was the dominant emitted terpenoid compound in both rivers, followed by camphene, β‐pinene, and junipene. In the BW river (Figure 3a), α‐pinene emissions were proportionally largest in summer (July–August) and lowest in spring (May–June). p‐cymene emissions showed an opposite temporal pattern to α‐pinene, with higher emissions in early summer than in late summer. Junipene emissions, the only observed SQT in the BW river, were proportionally higher during the early summers (Figure 3a). Emission profiles from the CW river showed no clear seasonal trends and displayed high variability throughout the years (Figure 3b). The most diverse compound profile was emitted in October 2022; out of 17 compounds detected in our campaigns, 14 were present in the CW river compared with 7 in the BW river. Camphene and β‐pinene contributed to the emission profiles consistently in both rivers, while p‐cymene and tricyclene were stable, contributing to the emission profiles in 2023.

### 3.3 Drivers of Emissions

**FIGURE 4:**
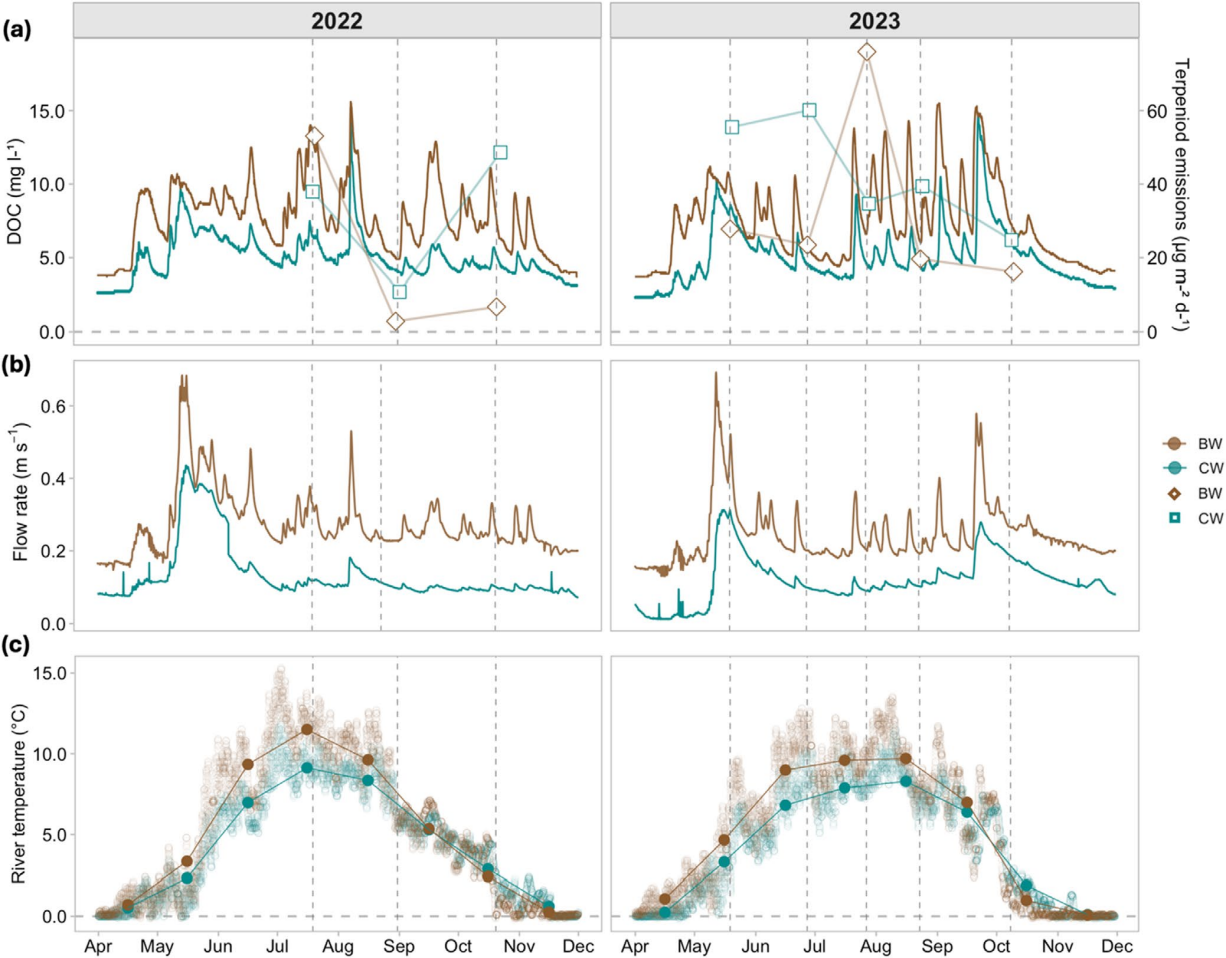
Continuous data from both rivers, (a) showing DOC concentration (mg L^−1^) from April to December in both rivers and the average terpenoid emissions as points (box and diamond shape for the brown water and clear water river, respectively); (b) showing flow rate (m s^−1^), and the (c) showing river temperature, with dots (•) displaying the monthly mean (°C). Sampling times are indicated by vertical dashed lines.

The BW river showed higher DOC concentration than the CW river, and DOC concentration varied with flow rate in both rivers (Figure 4a). The flow rate peaks in both rivers occurred concurrently, but the flow was lower and had less temporal variation in the CW river than in the BW river (Figure 4b). Occasionally, with an increase in DOC concentration, an increase in terpenoid emissions was also observed, particularly MT emissions (Figure 4a). In the BW river, DOC concentrations were high in July 2022 (16 ± 2 mg L^−1^) and July 2023 (8 ± 0.3 mg L^−1^) (Figure S2), and terpenoid emissions were also the highest during those months only. The aromatic characteristic of DOC, represented by SUVA_254_, exhibited different patterns. For the BW river in 2023, SUVA_254_ increased from summer until late autumn, whereas in 2022 it decreased. The CW river, on the other hand, showed a decreasing trend (Figure S3). The annual maximum flow rate peak was observed in May during the freshet, while the autumn peak varied, occurring in August in 2022 and October in 2023. Also, water temperature was higher in the BW river than in the CW river until September, after which the CW river had a higher temperature. The highest water temperatures in both rivers were measured in July, and the seasonal variability in temperature mostly followed PAR (Table S1, Figure 4c).

**FIGURE 5:**
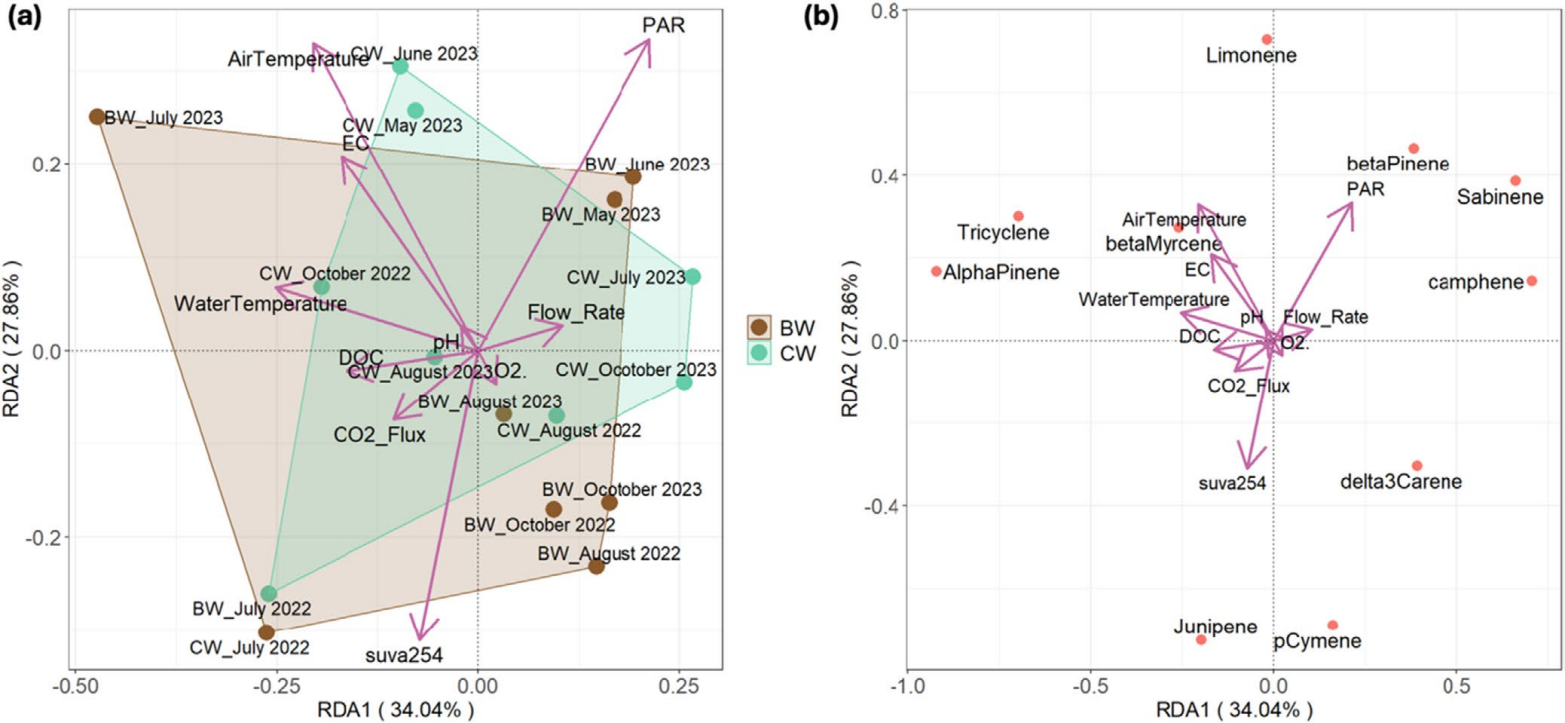
Redundancy analysis (RDA) ordination plots showing (a) the relationships between environmental variables and sampling times (*n* = 4) and (b) compounds. Arrows indicate the direction and magnitude of variables.

The differences in terpenoid emission profiles between the rivers and different sampling times and their relationships with environmental variables were analyzed using redundancy analysis (RDA). Seasonality rather than river influences the terpenoid emission profiles. The included variables explained 62% of the variation in terpenoid emissions among the two main components. The terpenoid emission peaks in summer (mainly comprised of MTs) were highly associated with temperatures, EC, DOC concentration, SUVA_254_, and CO_2_ flux, sorted in the negative extreme of the first component (Figure 5a). Terpenoid emissions during the early summers were positively correlated with PAR, except for the terpenoid emissions in July, when the emissions were influenced by flow rate. The ordination plot (Figure 5b) indicated a strong correlation between junipene, cymene, and SUVA_254_. Many MTs were positively correlated with temperature and PAR (Figure 5b).

We further evaluated the most influential variable(s) and their related coefficients on MT and SQT emissions using a multiple linear regression model (MLR) analysis (Table 1). MLR analysis showed that the best model for explaining MT emissions was model 10 (Table 1). Model 9 featured both river, air temperature, and river water DOC concentration as explanatory factors, explaining 64% of the variation in MT emissions. The MT emissions were most sensitive to changes in DOC concentration, while the effect of air temperature was not significant; see the simplified model (model 10), with only river and river water DOC concentration as explanatory variables.

For explaining SQT emissions, the model (model 8) included air temperature and SUVA_254_ as explanatory variables. It explained 60% of the variation in SQT emissions, 40% of which was explained by air temperature alone (Table 1). A further simplified model (model 9) shows a significant effect of air temperature on SQT emissions. More detailed MLR models are shown in Tables S2 and S3.

### 3.4 Emissions Estimated Over the Open‐Water Period

**FIGURE 6:**
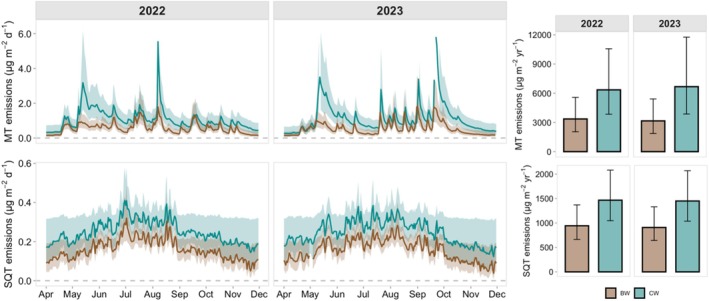
Total monoterpene (MT) and sesquiterpene (SQT) emissions estimated over the open‐water period using the MLR model 10 for MTs and model 9 for SQTs. The shadowed areas are error bars showing the 95% confidence interval of the estimated emissions. The bar graph shows the annual emissions of MT and SQT with error bars showing the 95% confidence interval.

We predicted the monthly and annual MT and SQT emissions using the best MLRs. The total MT emissions estimated for the BW river over the open‐water period were 3.4 ± 2.0 mg m^−2^ in 2022 and 3.2 ± 1.8 mg m^−2^ in 2023 and 6.3 ± 3.8 mg m^−2^ in 2022 and 6.7 ± 3.8 mg m^−2^ for the CW river in 2023, respectively (Figure 6). The estimated total SQT emissions were 0.9 ± 0.6 mg m^−2^ for the BW river in both 2022 and 2023 and 1.5 ± 1.0 mg m^−2^ for the CW river in both years.

## 4 Discussion

### 4.1 Emission Patterns and Composition

We compared MT and SQT emissions from two boreal rivers located in northern Finland in catchments with different soil and vegetation types over two growing seasons. Our study demonstrated that boreal rivers act as sources of terpenoids to the atmosphere. The overall emissions from the BW river, which was in a peatland catchment area, were lower compared to the CW river, which was in a mineral catchment area (1.2 ± 1.1 and 1.6 ± 1.1 μg m^−2^ h^−1^). In a study conducted near our sampling site in Värriö, Zhang‐Turpeinen et al. (2021) found that the average monthly forest floor emissions (per m^2^) were slightly higher than the river emissions reported here. They also observed similar compounds on the boreal forest floor as we detected from the river emissions (Zhang‐Turpeinen et al. 2021). The MT emissions from the river surface were slightly lower than the emissions from a minerotrophic fen in southern Finland (Männistö et al. 2023) and were lower than the emissions from the eutrophic ponds in a Danish peatland forest (Qin et al. 2025). Also, since the river is a flowing water system, its emissions could be lower because it has a shorter retention time, which reduces the accumulation of BVOCs compared to the stagnant conditions in ponds.

Vegetation and peat both contribute to terpenoid emissions in the boreal ecosystem (Männistö et al. 2023). *Vaccinium vitis‐idaea* , a common dwarf shrub species in boreal forests, has been shown to emit MTs (Vedel‐Petersen et al. 2015). Also, *Empetrum nigrum* and *Rhododendron tomentosum* , other common dwarf shrub species in this area, emit MTs and SQTs (Mofikoya et al. 2018; Schollert et al. 2015). During both years, the emissions in July were highest in the BW river, when maximum vegetation growth occurred. BVOCs synthesized by plants contribute to soil emissions through root exudates in the rhizosphere and leaf compounds stored in the litter (Peñuelas et al. 2014). Plant litter decomposition is another important source of BVOCs (Faubert et al. 2010; Wester‐Larsen et al. 2020). The BVOCs originating from litter in boreal forests are primarily composed of MTs (Aaltonen et al. 2011; Hakola et al. 2003), and this could also be reflected in the chemical composition of the water draining from these catchments, as the terpenoids can be released during decomposition.

The terpenoid emission patterns can also be influenced by changes in the activity of vegetation in the catchment areas and possibly by the productivity of aquatic plants. BVOC emissions from Scots pine ( *Pinus sylvestris* ) and other boreal species are partially associated with recent photosynthetic carbon assimilation (Ghirardo et al. 2010). The gross primary productivity (GPP) in both forest canopy and forest floor vegetation in Värriö forests peaks in early July and declines to zero by the end of October (Kulmala et al. 2019). The seasonal dynamics of GPP closely follow the intensity of PAR. We did not measure the GPP of aquatic vegetation in the rivers, but based on visual observation, the peak in the aquatic vegetation biomass occurred in July–August, as in the forest floor vegetation.

The mobility of BVOCs in soils and streams is primarily governed by their solubility. Beckmann and Lloyd (2001) reported that in bog peat profiles, BVOC concentrations in the water phase were substantially higher than in the gas phase; thus, the BVOC concentrations within peat, especially dissolved in water, may exceed atmospheric levels and be transported into rivers. Terpenoids, due to their hydrophobic and volatile nature, have a high kinetic potential to emit from surface water. Moreover, terpenoid emissions may be constrained by the high abundance of less degradable peatland‐derived DOM in the BW river (Saarela et al. 2024), or BVOCs can adhere to the organic matter in water and sediment (Rathbun 2000). Furthermore, lower emissions of SQTs compared to MTs could be attributed to their lower solubility and volatility, and less microbial transformation into other compounds due to their larger molecular size, resulting in greater retention (Copolovici and Niinemets 2015; Hui et al. 2023). This typically reduces their transfer from the water into the air, which could explain their lower overall emission rates.

Throughout both years and in both rivers, α‐pinene was the most abundant compound. Its dominance suggests a consistent and prolific source, whether from the surrounding terrestrial vegetation or from within the river's biological community. While its thermodynamic properties, such as its more negative solubility value (Δ*H*_sol_) (−21.0 kJ mol^−1^) compared to other MTs like β‐pinene (Δ*H*_sol_ −15.8 kJ mol^−1^) and myrcene (Δ*H*_sol_ −15.4 kJ mol^−1^) (Copolovici and Niinemets 2005), indicate that its solubility is highly sensitive to temperature fluctuations, the emissions of α‐pinene are not solely dependent on temperature. The absence of α‐pinene during the warmer months (June and July 2023) in the rivers (Figure 3) can suggest a different degradation pathway for DOC. This is further supported by the corresponding changes in SUVA_254_, PAR, and temperature conditions, suggesting that the response of the DOC degradation process to environmental factors during this period was different compared to other months.

Seasonality can be explained further by the seasonal changes in the hydrological flow paths within the peatland. The peat soil acts as a dynamic biogeochemical reservoir, with water flow shifting between different layers throughout the year (Holden 2006; Holden and Burt 2002; Jiao et al. 2023). During the wet seasons of spring and autumn, the high‐water table leads to significant surface flow through the upper, less decomposed layers of the peat. These layers, rich in organic matter and living moss, likely serve as the primary source for certain terpenoids. In the dry summer months, the water table drops. The dominant flow path shifts to the deeper, more decomposed peat layers and the underlying mineral soil. The extremely low hydraulic conductivity of this decomposed peat may limit the release of certain compounds, while the flow from the mineral soil acts as a chemical buffer, leading to a more stable composition of dissolved compounds. In contrast, the CW river showed less pronounced seasonal variability. The consistent flow from the underlying mineral soil provides a buffered and more stable chemical signature, which could partly explain the different temporal dynamics between these two catchments. The observed difference between the two rivers suggests a potential link between a catchment's unique physical and hydrological characteristics and the temporal dynamics of BVOC emissions.

Due to an absence of studies, BVOC sources within lotic ecosystems are not well known. Many MT compounds found in our observations, such as camphene, limonene, α‐pinene, and β‐pinene have been reported to be produced from phytoplankton (Meskhidze et al. 2015). The aquatic plants growing abundantly in marine ecosystems were reported to mainly release aldehydes, alkenes, alkanes, alcohols, arenes, ethers, furans, ketones, and phenol compounds. Microalgae in freshwater ecosystems were a source of VOCs containing sulphur, halogens, terpenoids, and oxygenated VOCs (Pozzer et al. 2022; Fink 2007; Peng et al. 2024). Algae and cyanobacteria are the primary producers of BVOCs, including terpenoids in aquatic systems (Saha and Fink 2022). However, the rivers in our study were oligotrophic, where the biomass of free‐living phytoplankton and microbes is extremely small, as indicated by the low turbidity values. (< 2) (Figure S5). Periphytic algae dominated these rivers, but to date, no information is available regarding the BVOC emissions of periphytic algae growing in subarctic environments.

### 4.2 Drivers of Emissions

In both rivers, DOC concentration was the most important factor explaining the MT emissions. DOC concentration was highly variable and responded to higher river flow rate and rain events. Water table increase is the most important variable explaining the DOC concentrations in peatlands (Rosset et al. 2020). In pristine peatland, as in our study, seasonal hydrological flow paths and precipitation events can raise the water table (Holden and Burt 2002) and flush DOM and BVOCs from peat decomposition and root exudation into the river, leading to greater seasonal variation in BVOC emissions from the BW river compared with the CW river.

Changes in air temperature are frequently seen as a key factor influencing DOC concentrations in peatland catchments (Billett et al. 2006; Clark et al. 2008; Dawson et al. 2011; Koehler et al. 2009). Higher temperatures directly increase DOC production by boosting plant and microbial activity in the terrestrial environment (Kalbitz et al. 2000; Pastor et al. 2003), with consequences to the DOC discharged from the soil to streams. In this study, as in an earlier study on the same rivers, they observed that spring‐exported DOM was highly aromatic and could be a source of VOCs (Saarela et al. 2024; Shaw et al. 2020). These differences in DOC concentration and quality can explain higher terpenoid emissions during the early summer than late summer in the BW river, as the higher SUVA_254_ values in spring indicated higher aromaticity (Figure S3). Although SUVA_254_ values remained high in August and October, the low degradability of peatland‐derived DOM (Saarela et al. 2024) was associated with reduced DOC concentrations, which could have led to decreased terpenoid emissions during late summer and early autumn.

However, overall higher terpenoid emissions were observed in the CW river, even though it had a lower DOC concentration than the BW river. This mismatch between spatial and temporal patterns of terpenoid emissions highlights the crucial role of DOC quality in addition to its quantity. Our MLR model confirms that while DOC concentration has a positive influence on MTs emissions within each river, the stronger determinant of overall emission rates is the inherent difference between the river types. Saarela et al. (2024) observed higher microbial degradation of DOM in the CW river compared to the BW river, which coincided with significantly lower aromaticity (SUVA_254_) in the CW river compared to the BW river. In addition, in the study of Saarela et al. (2024), the Fourier‐transform ion cyclotron resonance mass spectrometry (FTICR‐MS) measurements indicated a higher proportion of aliphatic and peptide‐like compounds in the CW river compared to the BW river, which is associated with high bioavailability and microbial activity in fluvial systems (Behnke et al. 2021; Begum et al. 2023). Also, the aliphatic‐rich signature in the CW river observed by Saarela et al. (2024) could indicate DOM of algal origin, which is associated with higher bioavailability compared to terrestrial or plant‐derived DOM (Guillemette et al. 2013), and it supports a greater growth efficiency of bacteria (Kritzberg et al. 2005). Higher aromaticity (i.e., SUVA_254_ values) indicates that the degradability of peatland‐derived DOM was more limited compared to mineral soil‐derived DOM (Saarela et al. 2024). The varying DOM composition and its susceptibility to breakdown between spring and autumn could be attributed to a shift in the primary source of DOM, from surface runoff during spring thaw to groundwater during baseflow conditions (Guo et al. 2007; Neff et al. 2006; Spencer et al. 2008).

Both air and water temperatures regulate DOC decomposition and BVOC solubility. Higher temperatures enhance microbial and enzymatic activity, accelerating organic substrate decomposition and increasing BVOC emissions like isoprene and monoterpenes (Peñuelas and Llusià 2001; Isidorov and Zaitsev 2022). Air temperature increases BVOC vapor pressure and volatility, facilitating their diffusion from plant tissues and soils to the atmosphere (Niinemets et al. 2010). Martins et al. (2017) showed an increase in the solubility of some terpenes with temperature. While the emissions of both terpene classes are temperature‐dependent, the effect is more apparent for SQTs, as shown by our MLR model. Firstly, SQTs have higher boiling points and lower volatility than MTs (Copolovici and Niinemets 2015). This means that a given increase in air temperature causes a much greater proportional increase in SQT volatility. Secondly, emissions from the river surface potentially originate from terpenoids produced by terrestrial and aquatic vegetation, as well as DOM inputs from the surrounding catchment. SQTs are typically not stored in large quantities in plants; instead, they are often produced via *de novo* synthesis in response to environmental stressors like temperature changes (Grote et al. 2019). This production process is highly temperature dependent, making it an important factor for SQT emissions. Lastly, the slow thermal response of a large river due to the high heat capacity of water means that air temperature may serve as a better proxy for the instantaneous thermal conditions influencing biological and chemical production in the surrounding catchment, from which a portion of these compounds likely originates. These processes suggest that warming stimulates BVOC production and release, limiting their retention in aqueous phases and potentially altering their transport and atmospheric reactivity.

Also, the turbulence has been shown to influence air–water gas exchange (Miettinen et al. 2015; Klaus et al. 2019). However, we did not observe any correlation between the terpenoid emissions and river water flow rate during the chamber measurements (BW: *p*‐value = 0.8; CW: *p*‐value = 0.9, Figure S6). Furthermore, according to our measurements, there was also no correlation between the terpenoid emissions and wind conditions either (BW: *p*‐value = 0.2; CW: *p*‐value = 0.4) (Figure S6), suggesting that the different seasonal patterns observed in the terpenoid emissions in these rivers were not related to these two factors. This could indicate that the differences between the emissions of these two rivers were more driven by aquatic terpenoid concentrations rather than differences in transfer velocities from river surface to atmosphere.

### 4.3 Emissions Estimated Over the Open‐Water Period

Global annual BVOC emissions are estimated to be 1200 Tg C using the MEGAN framework (Guenther et al. 2012). Global estimates of MT emissions range between 89 and 160 Tg C year^−1^, while SQT emissions are estimated at around 30 Tg C year^−1^ (Acosta Navarro et al. 2014). These estimates primarily account for terrestrial emissions, excluding contributions from aquatic ecosystems such as rivers and lakes. Using the MLR model, we estimated the river surface MT emission to range from 3.0 to 3.5 mg m^−2^ year^−1^ in the BW river and from 6.3 to 6.7 mg m^−2^ year^−1^ in the CW river, respectively, for 2 years. Similarly, the SQT emissions ranged from 1.0 mg m^−2^ year^−1^ in the BW river and 1.5 mg m^−2^ year^−1^ in the CW river. These findings represent one of the first attempts to quantify riverine emissions of terpenes, highlighting the importance of including aquatic systems in biogenic VOC budgets. To contextualize these results, we compared them with emissions reported from other ecosystems. For instance, modeled annual mean MT emissions from arctic plant ecosystems are approximately 8 mg C m^−2^ year^−1^, which is almost in a similar range to riverine estimates, suggesting river emissions are a significant source of BVOC emissions (Tang et al. 2016).

VOC emissions in a subarctic peatland and lake show that fens emitted higher levels of MTs during the growing season, while the lake primarily emitted methanol and acetone; no terpenoid emissions were reported from the lake (Seco et al. 2020). Their findings highlight the importance of both terrestrial and aquatic ecosystems in BVOC budgets, with fen emissions driven by temperature and light, and lake emissions linked to microbial activity (Seco et al. 2020). By contrast, forest floors emit a wide range of MTs and SQTs, with emissions varying significantly based on vegetation type and environmental conditions (Zhang‐Turpeinen et al. 2025). Forest floors have been shown to emit between 2% and 93% of the MT emissions observed from the canopy, depending on plant species. However, they were significantly lower than the summertime daytime MT emissions from the canopy of Scots pine trees in Hyytiälä forest, southern Finland, which have been reported to range from 60 to 100 ng m^−2^ s^−1^ (Rantala et al. 2015). This corresponds to approximately 220–360 μg m^−2^ h^−1^, two orders of magnitude higher than the emissions reported here. In the same forest, Aaltonen et al. (2011) measured MTs emission of 5.04 μg m^−2^ h^−1^ on the forest floor, which was in a similar magnitude as compared to our river emission. We found no other reports of terpenoid emissions from rivers.

Our observed total riverine MT emission rate of 4.9 ± 2.8 mg m^−2^ year^−1^ during the open‐water period falls within the emission range reported for forest floors and plant emissions and is lower than those observed in wetlands, permafrost‐affected peatland landscapes, and tundra ecosystems (Vettikkat et al. 2023; Hellén et al. 2020a, 2020b; Li et al. 2023; Jiao et al. 2023). Similarly, the SQT emissions of 1.2 ± 0.8 mg m^−2^ year^−1^ during the open‐water period are comparable to those reported from other ecosystems (Jiao et al. 2023; Duhl et al. 2008; Wang et al. 2017) and lower compared to the Amazon rainforest, vegetated pond, and thaw slumps (Bourtsoukidis et al. 2018; Jiao et al. 2023). These comparisons suggest that river surface emission rates are generally slightly lower or equal to boreal forest floor, eutrophic ponds, and arctic plant ecosystems but much lower than the emissions from Scots pine tree canopy. However, it is important to note that terpenoid emissions are influenced by factors such as vegetation type, temperature, light availability, and seasonal variations (Peñuelas and Staudt 2010; Vettikkat et al. 2023; Guenther et al. 1993; Niinemets et al. 2004; Laothawornkitkul et al. 2009; Peron et al. 2021). Therefore, direct comparisons across ecosystems should consider these variables to fully assess the contribution of rivers and other waterbodies to global BVOC budgets.

While our river emission rates per unit surface area were comparable to those reported for terrestrial ecosystems, it is crucial to consider the substantial disparity in global surface area between these systems. Rivers cover substantially smaller surface area than terrestrial ecosystems, approximately 0.6% of the global land surface area (Allen and Pavelsky 2018). When expressed per unit catchment area, riverine BVOC emissions are likely to be much smaller than terrestrial emissions. Therefore, when assessing the overall contribution of different ecosystems to global BVOC budgets, it is essential to account for their respective surface areas. Future research should focus on upscaling both river and terrestrial BVOC emissions to the catchment or regional scale to provide a more comprehensive understanding of their relative contributions.

## 5 Conclusions

This study highlights that rivers are a source of terpenoid emissions. Our results suggest that these emissions are controlled by a combination of factors that include the transport of compounds from terrestrial runoff and drainage, and the degradation of DOC within the rivers themselves. Furthermore, our findings indicate that river hydrology, water quality, and weather conditions play key roles in regulating both the quantity and chemical composition of terpenoid emissions. Additionally, our findings show that rivers with different catchments (peat vs. mineral) have distinct compound emission profiles. Using multiple linear regression modeling, we found that the drivers of terpenoid emissions are different for each class. MTs were primarily driven by DOC, while SQTs were driven by air temperature and SUVA_254_. Our study highlights the importance of including river emissions when estimating landscape‐level BVOC budgets, as aquatic systems can be a significant source. Furthermore, for a comprehensive assessment of their contribution to global BVOC budgets, emission comparisons across different ecosystems must also account for their respective surface areas. Our observations over 2 years from two rivers adjacent to peatland and mineral catchments provide the first estimate for terpenoid emissions from boreal rivers and the effects of weather conditions, as well as physicochemical and hydrological conditions in the rivers on these emissions. Given the importance of BVOCs in influencing atmospheric composition and subsequent events, there is an urgent need to conduct more extensive studies on rivers to formulate conclusions regarding the impact of the quantity and spatiotemporal distribution of BVOC emissions and sources on atmospheric chemistry and climate models.

## Author Contributions

**Wasi Hashmi:** data curation, formal analysis, investigation, methodology, software, validation, visualization, writing – original draft, writing – review and editing. **Huizhong Zhang‐Turpeinen:** investigation, methodology, supervision, visualization, writing – review and editing. **Lukas Kohl:** data curation, methodology, writing – review and editing. **Anne Kuningas:** investigation. **Carlos Palacin‐Lizarbe:** investigation, writing – review and editing. **Xudan Zhu:** data curation, writing – review and editing. **Niko Kinnunen:** investigation. **Maija E. Marushchak:** writing – review and editing. **Janne Rinne:** writing – review and editing. **Anne Ojala:** conceptualization, writing – review and editing. **Frank Berninger:** conceptualization, methodology, supervision, writing – review and editing. **Jukka Pumpanen:** conceptualization, funding acquisition, methodology, project administration, resources, supervision, writing – review and editing.

## Conflicts of Interest

The authors declare no conflicts of interest.

## Supporting information


**Figure S1:** The dominant aquatic plant species: (a) *Hippuris vulgaris* in the brown water river and (b) brook moss (*Fontinalis*) in the clear water river.
**Table S1:** The sampling dates and environmental conditions (i.e., river dissolved oxygen (O_2_%) concentrations, pH, electric conductivity, air temperature, and water temperature) were manually measured during the sampling occasions.
**Figure S2:** DOC concentration of water samples.
**Figure S3:** Suva_254_ concentration throughout the sampling campaign.
**Figure S4:** Meteorological data for the site from April 2022 to December 2023. Air temperature (°C), precipitation (mm day^−1^), PAR (μmol m^−2^ s^−1^), and humidity (%).
**Figure S5:** Turbidity continuous data from both rivers.
**Figure S6:** Regression line of terpenoid emission with flow rate (left) and wind speed (right).
**Table S2:** Final linear regression model of the monoterpene emissions, examining the relationship between environmental predictors and MT emissions.
**Table S3:** Final linear regression model of the sesquiterpene emissions, examining the relationship between environmental predictors and SQT emissions.
**Table S4:** Post hoc pairwise comparisons of month and years using Tukey's Honestly Significant Difference (HSD) test.

## Data Availability

The data that support the findings of this study are openly available at https://doi.org/10.6084/m9.figshare.28919453.

## References

[gcb70540-bib-0001] Aaltonen, H. , J. Pumpanen , M. Pihlatie , et al. 2011. “Boreal Pine Forest Floor Biogenic Volatile Organic Compound Emissions Peak in Early Summer and Autumn.” Agricultural and Forest Meteorology 151, no. 5–6: 682–691. 10.1016/j.agrformet.2010.12.010.

[gcb70540-bib-0002] Abis, L. , B. Loubet , R. Ciuraru , et al. 2018. “Profiles of Volatile Organic Compound Emissions From Soils Amended With Organic Waste Products.” Science of the Total Environment 636: 1333–1343. 10.1016/j.scitotenv.2018.04.232. 29913594

[gcb70540-bib-0003] Acosta Navarro, J. C. , S. Smolander , H. Struthers , et al. 2014. “Global Emissions of Terpenoid VOCs From Terrestrial Vegetation in the Last Millennium.” Journal of Geophysical Research: Atmospheres 119: 6867–6885. 10.1002/2013JD021238. PMC4370762 25866703

[gcb70540-bib-0004] Allen, G. H. , and T. M. Pavelsky . 2018. “Global Extent of Rivers and Streams.” Science 361: 585–588. 10.1126/science.aat0636. 29954985

[gcb70540-bib-0005] Baggesen, N. , T. Li , R. Seco , T. Holst , A. Michelsen , and R. Rinnan . 2021. “Phenological Stage of Tundra Vegetation Controls Bidirectional Exchange of BVOCs in a Climate Change Experiment on a Subarctic Heath.” Global Change Biology 27: 2928–2944. 10.1111/gcb.15596. 33709612 PMC8251604

[gcb70540-bib-0006] Bastviken, D. , I. Sundgren , S. Natchimuthi , H. Reyier , and M. Gålfalk . 2015. “Technical Note: Cost‐Efficient Approaches to Measure Carbon Dioxide (CO_2_) Fluxes and Concentrations in Terrestrial and Aquatic Environments Using Mini Loggers.” Biogeosciences 12: 3849–3859. 10.5194/bg-12-3849-2015.

[gcb70540-bib-0007] Battin, T. J. , R. Lauerwald , E. S. Bernhardt , et al. 2023. “River Ecosystem Metabolism and Carbon Biogeochemistry in a Changing World.” Nature 613: 449–459. 10.1038/s41586-022-05500-8. 36653564

[gcb70540-bib-0008] Beckmann, M. , and D. Lloyd . 2001. “Extraction and Identification of Volatile Organic Substances (VOS) From Scottish Peat Cores.” Atmospheric Environment 35: 79–86. 10.1016/S1352-2310(00)00290-9.

[gcb70540-bib-0009] Begum, M. S. , J. H. Park , L. Yang , K. H. Shin , and J. Hur . 2023. “Optical and Molecular Indices of Dissolved Organic Matter for Estimating Biodegradability and Resulting Carbon Dioxide Production in Inland Waters: A Review.” Water Research 228: 119362. 10.1016/j.watres.2022.119362. 36427460

[gcb70540-bib-0010] Behnke, M. I. , J. W. McClelland , S. E. Tank , et al. 2021. “Pan‐Arctic Riverine Dissolved Organic Matter: Synchronous Molecular Stability, Shifting Sources and Subsidies.” Global Biogeochemical Cycles 35: e2020GB006871. 10.1029/2020GB006871.

[gcb70540-bib-0011] Billett, M. F. , C. M. Deacon , S. M. Palmer , J. J. C. Dawson , and D. Hope . 2006. “Connecting Organic Carbon in Stream Water and Soils in a Peatland Catchment.” Journal of Geophysical Research 111: G02010. 10.1029/2005JG000065.

[gcb70540-bib-0012] Bourtsoukidis, E. , T. Behrendt , A. M. Yañez‐Serrano , et al. 2018. “Strong Sesquiterpene Emissions From Amazonian Soils.” Nature Communications 9, no. 1: 2226. 10.1038/s41467-018-04658-y. PMC5993744 29884892

[gcb70540-bib-0013] Boy, M. , P. Zhou , T. Kurtén , et al. 2022. “Positive Feedback Mechanism Between Biogenic Volatile Organic Compounds and the Methane Lifetime in Future Climates.” npj Climate and Atmospheric Science 5: 72. 10.1038/s41612-022-00292-0.

[gcb70540-bib-0014] Ciuraru, R. , L. Fine , M. van Pinxteren , B. D'Anna , H. Herrmann , and C. George . 2015. “Photosensitized Production of Functionalized and Unsaturated Organic Compounds at the Air‐Sea Interface.” Scientific Reports 5: 12741. 10.1038/srep12741. 26244712 PMC4650702

[gcb70540-bib-0015] Clark, J. M. , S. N. Lane , P. J. Chapman , and J. K. Adamson . 2008. “Link Between DOC in Near Surface Peat and Stream Water in an Upland Catchment.” Science of the Total Environment 404: 308–315. 10.1016/j.scitotenv.2007.11.002. 18076974

[gcb70540-bib-0016] Copolovici, L. , and Ü. Niinemets . 2015. “Temperature Dependencies of Henry's Law Constants for Different Plant Sesquiterpenes.” Chemosphere 138: 751–757. 10.1016/j.chemosphere.2015.07.075. 26291755 PMC5798578

[gcb70540-bib-0017] Copolovici, L. O. , and Ü. Niinemets . 2005. “Temperature Dependencies of Henry's Law Constants and Octanol/Water Partition Coefficients for Key Plant Volatile Monoterpenoids.” Chemosphere 61, no. 10: 1390–1400. 10.1016/j.chemosphere.2005.05.003. 15967478

[gcb70540-bib-0018] Dawson, J. J. C. , D. Tetzlaff , M. Speed , M. Hrachowitz , and C. Soulsby . 2011. “Seasonal Controls on DOC Dynamics in Nested Upland Catchments in NE Scotland.” Hydrological Processes 25: 1647–1658. 10.1002/hyp.7925.

[gcb70540-bib-0019] Delzer, G. C. , J. S. Zogorski , T. S. Lopes , and R. L. Bosshart . 1996. “Occurrence of the Gasoline Oxygenate MTBE and BTEX Compounds in Urban Stormwater in the United States, 1991‐95: U.S. Geological Survey Water Resources Investigations Report 96‐4145.” 6 p. 10.3133/wri964145.

[gcb70540-bib-0020] Duhl, T. R. , D. Helmig , and A. Guenther . 2008. “Sesquiterpene Emissions From Vegetation: A Review.” Biogeosciences 5: 761–777. 10.5194/bg-5-761-2008.

[gcb70540-bib-0021] Faubert, P. , P. Tiiva , Å. Rinnan , A. Michelsen , J. K. Holopainen , and R. Rinnan . 2010. “Doubled Volatile Organic Compound Emissions From Subarctic Tundra Under Simulated Climate Warming.” New Phytologist 187: 199–208. 10.1111/j.1469-8137.2010.03270.x. 20456056

[gcb70540-bib-0022] Feng, D. , C. J. Gleason , P. Lin , X. Yang , M. Pan , and Y. Ishitsuka . 2021. “Recent Changes to Arctic River Discharge.” Nature Communications 12: 6917. 10.1038/s41467-021-27228-1. PMC8617260 34824255

[gcb70540-bib-0023] Fichan, I. , C. Larroche , and J. B. Gros . 1998. “Water Solubility, Vapor Pressure, and Activity Coefficients of Terpenes and Terpenoids.” Journal of Chemical & Engineering Data 44, no. 1998: 56–62. 10.1021/je980070.

[gcb70540-bib-0024] Fink, P. 2007. “Ecological Functions of Volatile Organic Compounds in Aquatic Systems.” Marine and Freshwater Behaviour and Physiology 40, no. 3: 155–168. 10.1080/10236240701602218.

[gcb70540-bib-0025] Gålfalk, M. , D. Bastviken , S. Fredriksson , and L. Arneborg . 2013. “Determination of the Piston Velocity for Water‐Air Interfaces Using Flux Chambers, Acoustic Doppler Velocimetry, and IR Imaging of the Water Surface.” Journal of Geophysical Research – Biogeosciences 118: 770–782. 10.1002/jgrg.20064.

[gcb70540-bib-0026] Ghirardo, A. , K. Koch , R. Taipale , I. Zimmer , J.‐P. Schnitzler , and J. Rinne . 2010. “Determination of De Novo and Pool Emissions of Terpenes From Four Common Boreal/Alpine Trees by 13CO_2_ Labeling and PTR‐MS Analysis.” Plant, Cell & Environment 33: 781–792. 10.1111/j.1365-3040.2009.02104.x. 20040067

[gcb70540-bib-0027] Greenberg, J. P. , D. Asensio , A. Turnipseed , A. B. Guenther , T. Karl , and D. Gochis . 2012. “Contribution of Leaf and Needle Litter to Whole Ecosystem BVOC Fluxes.” Atmospheric Environment 59: 302–311. 10.1016/j.atmosenv.2012.04.038.

[gcb70540-bib-0028] Grote, R. , M. Sharma , A. Ghirardo , and J. P. Schnitzler . 2019. “A New Modeling Approach for Estimating Abiotic and Biotic Stress‐Induced De Novo Emissions of Biogenic Volatile Organic Compounds From Plants.” Frontiers in Forests and Global Change 2: 26. 10.3389/ffgc.2019.00026.

[gcb70540-bib-0029] Guenther, A. , C. N. Hewitt , D. Erickson , et al. 1995. “A Global Model of Natural Volatile Organic Compound Emissions.” Journal of Geophysical Research: Atmospheres 100, no. D5: 8873–8892. 10.1029/94JD02950.

[gcb70540-bib-0030] Guenther, A. B. , X. Jiang , C. L. Heald , et al. 2012. “The Model of Emissions of Gases and Aerosols From Nature Version 2.1 (MEGAN2.1): An Extended and Updated Framework for Modeling Biogenic Emissions.” Geoscientific Model Development 5, no. 6: 1471–1492. 10.5194/gmd-5-1471-2012.

[gcb70540-bib-0031] Guenther, A. B. , P. R. Zimmerman , P. C. Harley , R. K. Monson , and R. Fall . 1993. “Isoprene and Monoterpene Emission Rate Variability: Model Evaluations and Sensitivity Analyses.” Journal of Geophysical Research: Atmospheres 98, no. D7: 12609–12617. 10.1029/93jd00527.

[gcb70540-bib-0032] Guillemette, F. , S. L. McCallister , and P. A. del Giorgio . 2013. “Differentiating the Degradation Dynamics of Algal and Terrestrial Carbon Within Complex Natural Dissolved Organic Carbon in Temperate Lakes.” Journal of Geophysical Research: Biogeosciences 118, no. 3: 963–973. 10.1002/jgrg.20077.

[gcb70540-bib-0033] Guo, L. , C.‐L. Ping , and R. W. Macdonald . 2007. “Mobilization Pathways of Organic Carbon From Permafrost to Arctic Rivers in a Changing Climate.” Geophysical Research Letters 34: L13603. 10.1029/2007GL030689.

[gcb70540-bib-0034] Hakola, H. , V. Tarvainen , T. Laurila , V. Hiltunen , H. Hellen , and P. Keronen . 2003. “Seasonal Variation of VOC Concentrations Above a Boreal Coniferous Forest.” Atmospheric Environment 37, no. 12: 1623–1634. 10.1016/S1352-2310(03)00014-1.

[gcb70540-bib-0036] Hellén, H. , S. Schallhart , A. P. Praplan , et al. 2020a. “Sesquiterpenes Dominate Monoterpenes in Northern Wetland Emissions.” Atmospheric Chemistry and Physics 20: 7021–7034. 10.5194/acp-20-7021-2020.

[gcb70540-bib-0037] Hellén, H. , S. Schallhart , A. P. Praplan , et al. 2020b. “Supplement to: Sesquiterpenes Dominate Monoterpenes in Northern Wetland Emissions.” Atmospheric Chemistry and Physics 20, no. 11: 7021–7034. 10.5194/acp-20-7021-2020-supplement.

[gcb70540-bib-0038] Holden, J. 2006. “Chapter 14 Peatland Hydrology.” In Developments in Earth Surface Processes, vol. 9, 319–346. Elsevier. 10.1016/S0928-2025(06)09014-6.

[gcb70540-bib-0039] Holden, J. , and T. P. Burt . 2002. “Infiltration, Runoff and Sediment Production in Blanket Peat Catchments: Implications of Field Rainfall Simulation Experiments.” Hydrological Processes 16: 2537–2557. 10.1002/hyp.1014.

[gcb70540-bib-0040] Holden, J. , and T. P. Burt . 2003. “Runoff Production in Blanket Peat Covered Catchments.” Water Resources Research 39: 1191. 10.1029/2002WR001956.

[gcb70540-bib-0041] Hui, K. , Y. Yuan , B. Xi , and W. Tan . 2023. “A Review of the Factors Affecting the Emission of the Ozone Chemical Precursors VOCs and NOx From the Soil.” Environment International 172: 107799. 10.1016/j.envint.2023.107799. 36758299

[gcb70540-bib-0042] Isidorov, V. A. , and A. A. Zaitsev . 2022. “Reviews and Syntheses: VOC Emissions From Soil Cover in Boreal and Temperate Natural Ecosystems of the Northern Hemisphere.” Biogeosciences 19: 4715–4746. 10.5194/bg-19-4715-2022.

[gcb70540-bib-0043] Isidorov, V. A. , I. G. Zenkevich , and B. V. Ioffe . 1985. “Volatile Organic Compounds in the Atmosphere of Forests.” Atmospheric Environment (1967) 19: 1–8. 10.1016/0004-6981(85)90131-3.

[gcb70540-bib-0044] Ivanov, K. E. 1981. Water Movement in Mirelands. Academic Press. 10.1177/030913338300700409.

[gcb70540-bib-0045] Jiao, Y. , C. L. Davie‐Martin , M. Kramshøj , et al. 2023. “Volatile Organic Compound Release Across a Permafrost‐Affected Peatland.” Geoderma 430: 116355. 10.1016/j.geoderma.2023.116355.

[gcb70540-bib-0046] Kalbitz, K. , S. Solinger , J. H. Park , B. Michalzik , and E. Matzner . 2000. “Controls on the Dynamics of Dissolved Organic Matter in Soils: A Review.” Soil Science 165: 277–304. 10.1097/00010694-200004000-00001.

[gcb70540-bib-0047] Kesselmeier, J. , and M. Staudt . 1999. “Biogenic Volatile Organic Compounds (VOC): An Overview on Emission, Physiology and Ecology.” Journal of Atmospheric Chemistry 33, no. 1: 23–88. 10.1023/A:1006127516791.

[gcb70540-bib-0048] Klaus, M. , E. Geibrink , E. R. Hotchkiss , and J. Karlsson . 2019. “Listening to Air–Water Gas Exchange in Running Waters.” Limnology and Oceanography: Methods 17: 395–414. 10.1002/lom3.10321.

[gcb70540-bib-0049] Koehler, A. K. , K. Murphy , G. Kiely , and M. Sottocornola . 2009. “Seasonal Variation of DOC Concentration and Annual Loss of DOC From an Atlantic Blanket Bog in South Western Ireland.” Biogeochemistry 95: 231–242. 10.1007/s10533-009-9333-9.

[gcb70540-bib-0050] Kritzberg, E. , J. J. Cole , M. M. Pace , and W. Granéli . 2005. “Does Autochthonous Primary Production Drive Variability in Bacterial Metabolism and Growth Efficiency in Lakes Dominated by Terrestrial C Inputs?” Aquatic Microbial Ecology 38, no. 2: 103–111. http://www.int‐res.com/abstracts/ame/v38/n2/p103‐111/.

[gcb70540-bib-0051] Kulmala, L. , J. Pumpanen , P. Kolari , et al. 2019. “Inter‐ and Intra‐Annual Dynamics of Photosynthesis Differ Between Forest Floor Vegetation and Tree Canopy in a Subarctic Scots Pine Stand.” Agricultural and Forest Meteorology 271: 1–11. 10.1016/j.agrformet.2019.02.029.

[gcb70540-bib-0052] Laothawornkitkul, J. , J. E. Taylor , N. D. Paul , and C. N. Hewitt . 2009. “Biogenic Volatile Organic Compounds in the Earth System.” New Phytologist 183: 27–51. 10.1111/j.1469-8137.2009.02859.x. 19422541

[gcb70540-bib-0053] Li, T. , N. Baggesen , R. Seco , and R. Rinnan . 2023. “Seasonal and Diel Patterns of Biogenic Volatile Organic Compound Fluxes in a Subarctic Tundra.” Atmospheric Environment 292: 119430. 10.1016/j.atmosenv.2022.119430.

[gcb70540-bib-0054] Lin, C. , S. M. Owen , and J. Peñuelas . 2007. “Volatile Organic Compounds in the Roots and Rhizosphere of *Pinus* spp.” Soil Biology & Biochemistry 39: 951–960. 10.1016/j.soilbio.2006.11.007.

[gcb70540-bib-0055] Lindwall, F. , M. Schollert , A. Michelsen , D. Blok , and R. Rinnan . 2016. “Fourfold Higher Tundra Volatile Emissions due to Arctic Summer Warming.” Journal of Geophysical Research – Biogeosciences 121, no. 3: 895–902. 10.1002/2015JG003295.

[gcb70540-bib-0056] Männistö, E. , H. Ylanne , M. Losoi , et al. 2023. “Emissions of Biogenic Volatile Organic Compounds From Adjacent Boreal Fen and Bog as Impacted by Vegetation Composition.” Science of the Total Environment 858: 159809. 10.1016/j.scitotenv.2022.159809. 36336039

[gcb70540-bib-0057] Martins, M. A. R. , L. P. Silva , O. Ferreira , B. Schröder , J. A. P. Coutinho , and S. P. Pinho . 2017. “Terpenes Solubility in Water and Their Environmental Distribution.” Journal of Molecular Liquids 241: 996–1002. 10.1016/j.molliq.2017.06.099.

[gcb70540-bib-0058] Meskhidze, N. , A. Sabolis , R. Reed , and D. Kamykowski . 2015. “Quantifying Environmental Stress‐Induced Emissions of Algal Isoprene and Monoterpenes Using Laboratory Measurements.” Biogeosciences 12: 637–651. 10.5194/bg-12-637-2015.

[gcb70540-bib-0059] Miettinen, H. , J. Pumpanen , J. J. Heiskanen , et al. 2015. “Towards a More Comprehensive Understanding of Lacustrine Greenhouse Gas Dynamics Two‐Year Measurements of Concentrations and Fluxes of CO_2_, CH_4_ and N_2_O in a Typical Boreal Lake Surrounded by Managed Forests.” Boreal Environment Research 20: 75–89. http://hdl.handle.net/10138/165214.

[gcb70540-bib-0060] Mofikoya, A. O. , K. Miura , R. P. Ghimire , et al. 2018. “Understorey *Rhododendron tomentosum* and Leaf Trichome Density Affect Mountain Birch VOC Emissions in the Subarctic.” Scientific Reports 8: 13261. 10.1038/s41598-018-31084-3. 30185795 PMC6125604

[gcb70540-bib-0061] Neff, J. C. , J. C. Finlay , S. A. Zimov , et al. 2006. “Seasonal Changes in the Age and Structure of Dissolved Organic Carbon in Siberian Rivers and Streams, Geophysical.” Research Letters 33: L23401. 10.1029/2006GL028222.

[gcb70540-bib-0062] Niinemets, Ü. , A. Arneth , U. Kuhn , R. K. Monson , J. Peñuelas , and M. Staudt . 2010. “The Emission Factor of Volatile Isoprenoids: Stress, Acclimation, and Developmental Responses.” Biogeosciences 7: 2203–2223. 10.5194/bg-7-2203-2010.

[gcb70540-bib-0063] Niinemets, Ü. , F. Loreto , and M. Reichstein . 2004. “Physiological and Physicochemical Controls on Subsistence of Volatile Organic Compounds From Leaves.” Trends in Plant Science 9: 180–186. 10.1016/j.tplants.2004.02.006. 15063868

[gcb70540-bib-0064] Olefeldt, D. , N. Roulet , R. Giesler , and A. Persson . 2013. “Total Waterborne Carbon Export and DOC Composition From Ten Nested Subarctic Peatland Catchments—Importance of Peatland Cover, Groundwater Influence, and Inter‐Annual Variability of Precipitation Patterns.” Hydrological Processes 27: 2280–2294. 10.1002/hyp.9358.

[gcb70540-bib-0065] Pastor, J. , J. Solin , S. D. Bridgham , et al. 2003. “Global Warming and the Export of Dissolved Organic Carbon From Boreal Peatlands.” Oikos 100, no. 2: 380–386. http://www.jstor.org/stable/3548196.

[gcb70540-bib-0066] Peng, Q. , Y. Yang , W. Ou , et al. 2024. “The Characteristics and Environmental Significance of BVOCs Released by Aquatic Macrophytes.” Chemosphere 361: 142574. 10.1016/j.chemosphere.2024.142574. 38852633

[gcb70540-bib-0067] Peñuelas, J. , D. Asensio , D. Tholl , et al. 2014. “Biogenic Volatile Emissions From the Soil.” Plant, Cell & Environment 37: 1866–1891. 10.1111/pce.12340. 24689847

[gcb70540-bib-0068] Peñuelas, J. , and J. Llusià . 2001. “The Complexity of Factors Driving Volatile Organic Compound Emissions by Plants.” Biologia Plantarum 44: 481–487. 10.1023/A:1013797129428.

[gcb70540-bib-0069] Peñuelas, J. , and M. Staudt . 2010. “BVOCs and Global Change.” Trends in Plant Science 15, no. 3: 133–144. 10.1016/j.tplants.2009.12.005. 20097116

[gcb70540-bib-0070] Peron, A. , L. Kaser , A. C. Fitzky , et al. 2021. “Combined Effects of Ozone and Drought Stress on the Emission of Biogenic Volatile Organic Compounds From *Quercus robur* L.” Biogeosciences 18: 535–556. 10.5194/bg-18-535-2021.

[gcb70540-bib-0071] Pozzer, A. C. , P. A. Gómez , and J. Weiss . 2022. “Volatile Organic Compounds in Aquatic Ecosystems ‐ Detection, Origin, Significance and Applications.” Science of the Total Environment 838: 156155. 10.1016/j.scitotenv.2022.156155. 35609693

[gcb70540-bib-0072] Qin, Y. , K. E. Roslund , A. S. Smart , J. R. Christiansen , and R. Rinnan . 2025. “Net Emission of Atmospheric Volatile Organic Compounds From Ponds in a Peatland Forest.” Limnology and Oceanography. 10.1002/lno.70202.

[gcb70540-bib-0073] Rantala, P. , J. Aalto , R. Taipale , T. M. Ruuskanen , and J. Rinne . 2015. “Annual Cycle of Volatile Organic Compound Exchange Between a Boreal Pine Forest and the Atmosphere.” Biogeosciences 12, no. 19: 5753–5770. 10.5194/bg-12-5753-2015.

[gcb70540-bib-0074] Rathbun, R. E. 2000. “Transport, Behavior, and Fate of Volatile Organic Compounds in Streams.” Critical Reviews in Environmental Science and Technology 30, no. 2: 129–295. 10.1080/10643380091184183.

[gcb70540-bib-0075] Regnier, P. , L. Resplandy , R. G. Najjar , and P. Ciais . 2022. “The Land‐to‐Ocean Loops of the Global Carbon Cycle.” Nature 603: 401–410. 10.1038/s41586-021-04339-9. 35296840

[gcb70540-bib-0076] Rosset, T. , S. Binet , J.‐M. Antoine , E. Lerigoleur , F. Rigal , and L. Gandois . 2020. “Drivers of Seasonal‐ and Event‐Scale DOC Dynamics at the Outlet of Mountainous Peatlands Revealed by High‐Frequency Monitoring.” Biogeosciences 17: 3705–3722. 10.5194/bg-17-3705-2020.

[gcb70540-bib-0077] Saarela, T. , X. Zhu , T. Kekäläinen , et al. 2024. “The Influence of Dissolved Organic Matter Composition on Microbial Degradation and Carbon Dioxide Production in Pristine Subarctic Rivers.” Biogeochemistry 29: 131–148. https://www.borenv.net/BER/archive/pdfs/ber29/ber29‐131‐148.pdf.

[gcb70540-bib-0078] Saha, M. , and P. Fink . 2022. “Algal Volatiles – The Overlooked Chemical Language of Aquatic Primary Producers.” Biological Reviews 97: 2162–2173. 10.1111/brv.12887. 35912802

[gcb70540-bib-0079] Sakulyanontvittaya, T. , T. Duhl , C. Wiedinmyer , et al. 2008. “Monoterpene and Sesquiterpene Emission Estimates for the United States.” Environmental Science & Technology 42, no. 5: 1623–1629. 10.1021/es702274e. 18441812

[gcb70540-bib-0080] Schindler, D. W. 1998. “Sustaining Aquatic Ecosystems in Boreal Regions.” Conservation Ecology 2, no. 2: 18. http://www.consecol.org/vol2/iss2/art18/.

[gcb70540-bib-0081] Schollert, M. , M. Kivimäenpää , H. M. Valolahti , and R. Rinnan . 2015. “Climate Change Alters Leaf Anatomy, but Has no Effects on Volatile Emissions From Arctic Plants.” Plant, Cell & Environment 38: 2048–2060. 10.1111/pce.12530. 25737381

[gcb70540-bib-0082] Seco, R. , T. Holst , M. S. Matzen , et al. 2020. “Volatile Organic Compound Fluxes in a Subarctic Peatland and Lake.” Atmospheric Chemistry and Physics 20: 13399–13416. 10.5194/acp-20-13399-2020.

[gcb70540-bib-0083] Shaw, J. T. , A. R. Rickard , M. J. Newland , and T. J. Dillon . 2020. “Rate Coefficients for Reactions of OH With Aromatic and Aliphatic Volatile Organic Compounds Determined by the Multivariate Relative Rate Technique.” Atmospheric Chemistry and Physics 20: 9725–9736. 10.5194/acp-20-9725-2020.

[gcb70540-bib-0084] Spencer, R. G. M. , G. R. Aiken , K. P. Wickland , R. G. Striegl , and P. J. Hernes . 2008. “Seasonal and Spatial Variability in Dissolved Organic Matter Quantity and Composition From the Yukon River Basin, Alaska.” Global Biogeochemical Cycles 22: GB4002. https://agupubs.onlinelibrary.wiley.com/doi/pdf/10.1029/2008GB003231.

[gcb70540-bib-0085] Svendsen, S. H. , A. Priemé , J. Voriskova , et al. 2018. “Emissions of Biogenic Volatile Organic Compounds From Arctic Shrub Litter Are Coupled With Changes in the Bacterial Community Composition.” Soil Biology and Biochemistry 120: 80–90. 10.1016/j.soilbio.2018.02.001.

[gcb70540-bib-0086] Tang, J. , G. Schurgers , and R. Rinnan . 2019. “Process Understanding of Soil BVOC Fluxes in Natural Ecosystems: A Review.” Reviews of Geophysics 57: 966–986. 10.1029/2018RG000634.

[gcb70540-bib-0087] Tang, J. , G. Schurgers , H. Valolahti , et al. 2016. “Challenges in Modelling Isoprene and Monoterpene Emission Dynamics of Arctic Plants: A Case Study From a Subarctic Tundra Heath.” Biogeosciences 13: 6651–6667. 10.5194/bg-13-6651-2016.

[gcb70540-bib-0088] Terracciano, S. , and A. O'Brien . 1997. Occurrence of Volatile Organic Compounds in Streams on Long Island, New York, and New Jersey Overview of Available Data and Reconnaissance Sampling. Geological Survey. 10.3133/fs06397.

[gcb70540-bib-0089] Vedel‐Petersen, I. , M. Schollert , J. Nymand , and R. Rinnan . 2015. “Volatile Organic Compound Emission Profiles of Four Common Arctic Plants.” Atmosphere Environment 120: 117–126. 10.1016/j.atmosenv.2015.08.082.

[gcb70540-bib-0090] Vettikkat, L. , P. Miettinen , A. Buchholz , et al. 2023. “High Emission Rates and Strong Temperature Response Make Boreal Wetlands a Large Source of Isoprene and Terpenes.” Atmospheric Chemistry and Physics 23: 2683–2698. 10.5194/acp-23-2683-2023.

[gcb70540-bib-0091] Wang, C. , W. Wang , X. Liu , et al. 2024. “Aqueous VOCs in Complex Water Environment of Oil Exploitation Sites: Spatial Distribution, Migration Flux, and Risk Assessment.” Journal of Hazardous Materials 476: 135121. 10.1016/j.jhazmat.2024.135121. 38981233

[gcb70540-bib-0092] Wang, H. , X. Liu , C. Wu , and G. Lin . 2024. “Regional to Global Distributions, Trends, and Drivers of Biogenic Volatile Organic Compound Emission From 2001 to 2020.” Atmospheric Chemistry and Physics 24: 3309–3328. 10.5194/acp-24-3309-2024.

[gcb70540-bib-0093] Wang, M. , G. Schurgers , A. Arneth , A. Ekberg , and T. Holst . 2017. “Seasonal Variation in Biogenic Volatile Organic Compound (BVOC) Emissions From Norway Spruce in a Swedish Boreal Forest.” Boreal Environment Research 22: 353–367. 10.60910/b4g6-6zs5.

[gcb70540-bib-0094] Weidenhamer, J. D. , F. A. Macias , N. H. Fischer , and G. B. Williamson . 1993. “Just How Insoluble Are Monoterpenes?” Journal of Chemical Ecology 19: 1799–1807. 10.1007/BF00982309. 24249242

[gcb70540-bib-0095] Wester‐Larsen, L. , M. Kramshøj , C. N. Albers , and R. Rinnan . 2020. “Biogenic Volatile Organic Compounds in Arctic Soil: A Field Study of Concentrations and Variability With Vegetation Cover.” Journal of Geophysical Research: Biogeosciences 125: e2019JG005551. 10.1029/2019JG005551.

[gcb70540-bib-0096] Wrona, F. J. , M. Johansson , J. M. Culp , et al. 2016. “Transitions in Arctic Ecosystems: Ecological Implications of a Changing Hydrological Regime.” Journal of Geophysical Research: Biogeosciences 121, no. 3: 650–674. 10.1002/2015JG003133.

[gcb70540-bib-0097] Zhang‐Turpeinen, H. , H. Aaltonen , J. Tang , et al. 2025. “Changes in the Factors Influencing Forest Floor Terpenoid Emissions During Post‐Fire Forest Succession.” Journal of Geophysical Research: Biogeosciences 130: e2024JG008113. 10.1029/2024JG008113.

[gcb70540-bib-0098] Zhang‐Turpeinen, H. , M. Kivimäenpää , H. Aaltonen , et al. 2020. “Wildfire Effects on BVOC Emissions From Boreal Forest Floor on Permafrost Soil in Siberia.” Science of the Total Environment 711: 134851. 10.1016/j.scitotenv.2019.134851. 32000328

[gcb70540-bib-0099] Zhang‐Turpeinen, H. , M. Kivimäenpää , F. Berninger , et al. 2021. “Age‐Related Response of Forest Floor Biogenic Volatile Organic Compound Fluxes to Boreal Forest Succession After Wildfires.” Agricultural and Forest Meteorology 308: 108584. 10.1016/j.agrformet.2021.108584.

[gcb70540-bib-0100] Zhao, D. , Y. Yang , Y. J. Tham , and S. Zou . 2023. “Emission of Marine Volatile Organic Compounds (VOCs) by Phytoplankton ‐ A Review.” Marine Environmental Research 191: 106177. 10.1016/j.marenvres.2023.106177. 37741052

[gcb70540-bib-0101] Zhou, S. , L. Gonzalez , A. Leithead , et al. 2014. “Formation of Gas‐Phase Carbonyls From Heterogeneous Oxidation of Polyunsaturated Fatty Acids at the Air‐Water Interface and of the Sea Surface Microlayer.” Atmospheric Chemistry and Physics 14, no. 3: 1371–1384. 10.5194/acp-14-1371-2014.

[gcb70540-bib-0102] Zhu, X. , L. Chen , J. Pumpanen , et al. 2021. “Assessment of a Portable UV–vis Spectrophotometer's Performance for Stream Water DOC and Fe Content Monitoring in Remote Areas.” Talanta 224: 121919. 10.1016/j.talanta.2020.121919. 33379120

[gcb70540-bib-0103] Zhu, X. , L. Chen , J. Pumpanen , et al. 2022. “The Role of Terrestrial Productivity and Hydrology in Regulating Aquatic Dissolved Organic Carbon Concentrations in Boreal Catchments.” Global Change Biology 28, no. 8: 2764–2778. 10.1111/gcb.16094. 35060250 PMC9303698

