# Supplementary material for: Boreal Rivers as Sources of Terpenoid Emissions
Source: Glob Chang Biol. 2025 Oct 10;31(10):e70540. doi: 10.1111/gcb.70540 (PMC12512008; doi:10.1111/gcb.70540)
Supplement: Supplementary file 1 — Figure S1: The dominant aquatic plant species: (a) Hippuris vulgaris in the brown water river and (b) brook moss (Fontinalis) in the clear water river. Table S1: The sampling dates and environmental conditions (i.e., river dissolved oxygen (O2%) concentrations, pH, electric conductivity, air temperature, and water temperature) were manually measured during the sampling occasions. Figure S2: DOC concentration of water samples. Figure S3: Suva254 concentration throughout the sampling campaign. Figure S4: Meteorological data for the site from April 2022 to December 2023. Air temperature (°C), precipitation (mm day−1), PAR (μmol m−2 s−1), and humidity (%). Figure S5: Turbidity continuous data from both rivers. Figure S6: Regression line of terpenoid emission with flow rate (left) and wind speed (right). Table S2: Final linear regression model of the monoterpene emissions, examining the relationship between environmental predictors and MT emissions. Table S3: Final linear regression model of the sesquiterpene emissions, examining the relationship between environmental predictors and SQT emissions. Table S4: Post hoc pairwise comparisons of month and years using Tukey's Honestly Significant Difference (HSD) test. [file GCB-31-e70540-s001.docx]

*Journal of Global Change Biology*

Supporting Information for

**Boreal rivers as sources of terpenoid emissions**

Wasi Hashmi^1^, Huizhong Zhang-Turpeinen^1^, Lukas Kohl^1^, Anne Kuningas^1^, Carlos Palacin-Lizarbe^1^, Xudan Zhu^2^, Niko Kinnunen^3^, Maija E. Marushchak^1^, Janne Rinne^3^, Anne Ojala^3^, Frank Berninger^2^, Jukka Pumpanen^1^

^1^ Department of Environmental and Biological Sciences, University of Eastern Finland, Kuopio, FI-70210, Finland.

^2^ Department of Environmental and Biological Sciences, University of Eastern Finland, Joensuu, FI-80101, Finland.

^3^ Natural Resources Institute Finland, (Luke), Helsinki, Uusima, Finland

**Contents of this file**

Figures S1 to S6

Tables S1 to S4


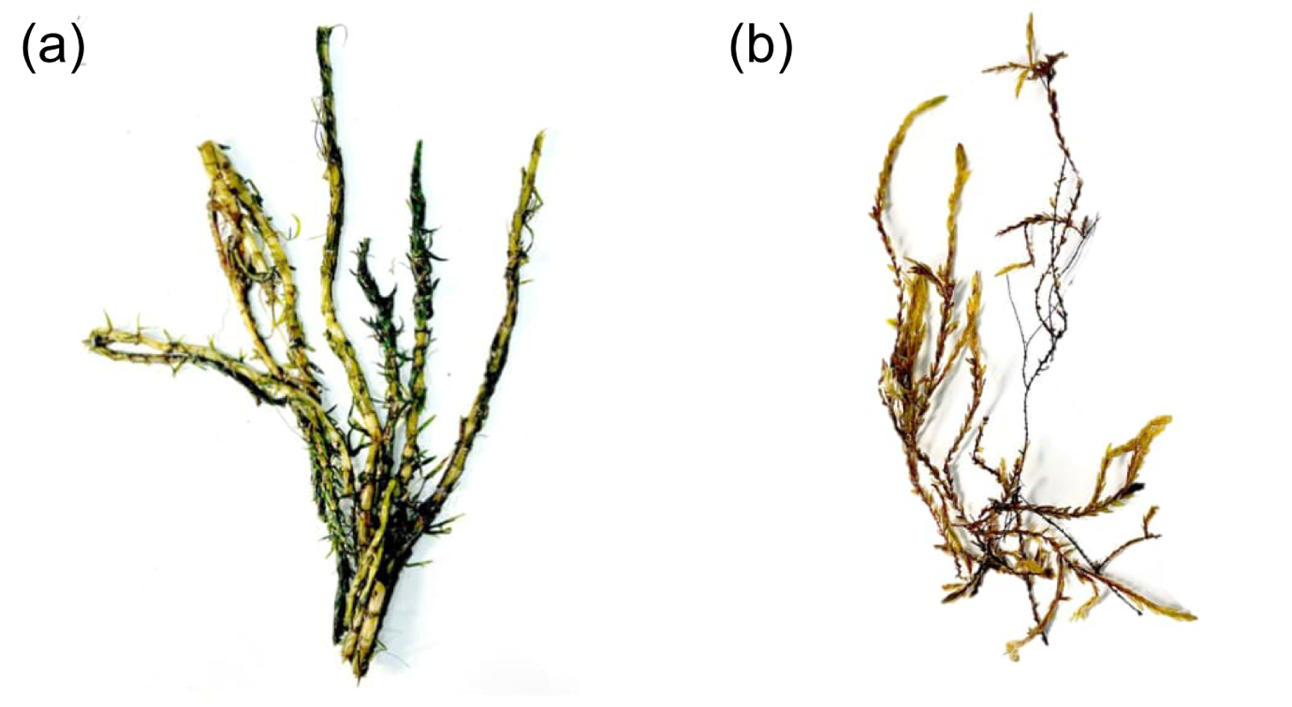


**Figure S1.** The dominant aquatic plant species: (a) Hippuris vulgaris in the brown water river and (b) brook moss (*Fontinalis*) in the clear water river.

**Table S1.** The sampling dates and environmental conditions (i.e., river dissolved oxygen (O2%) concentrations, pH, electric conductivity, air temperature, and water temperature) were manually measured during the sampling occasions. NA = not analysed.


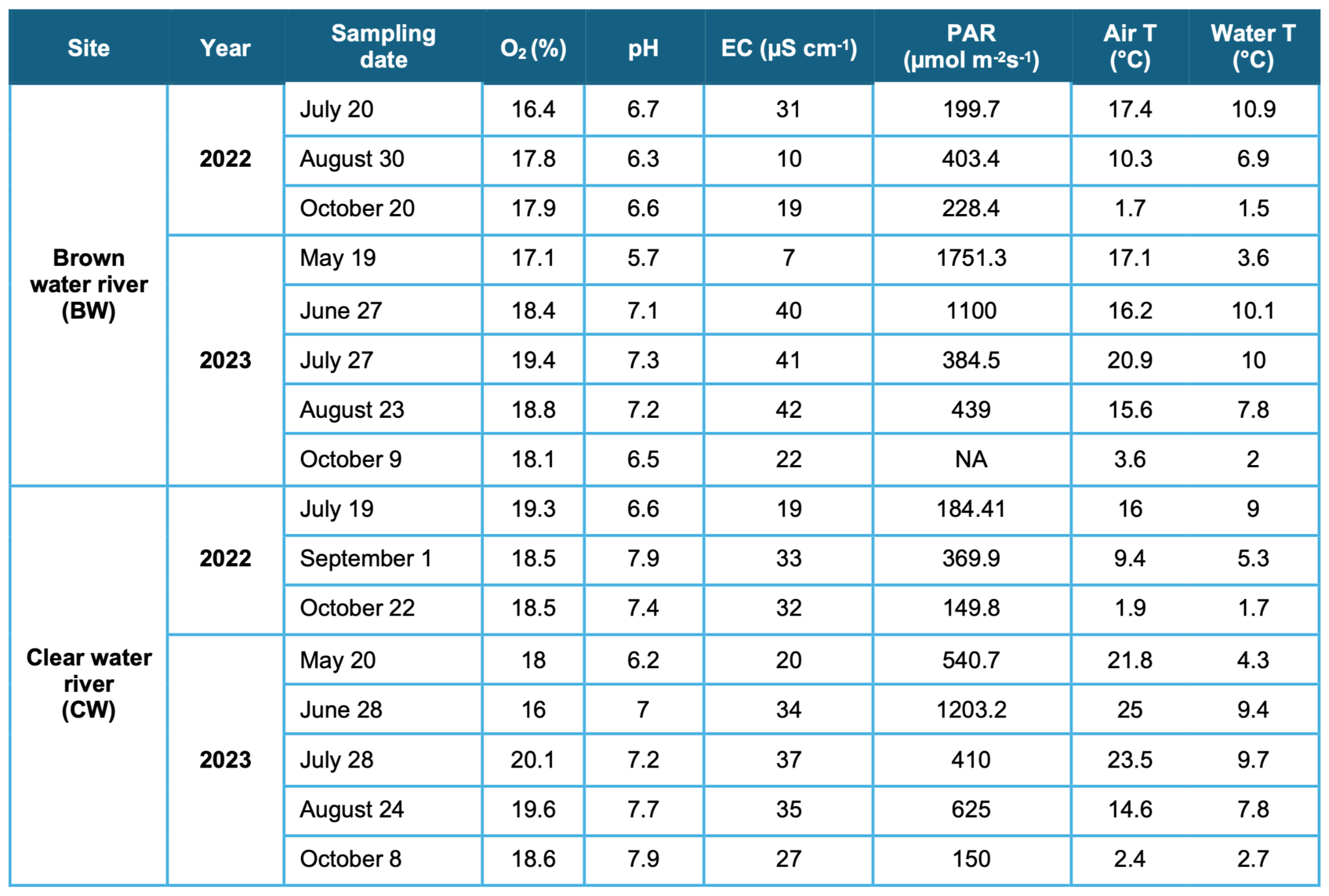


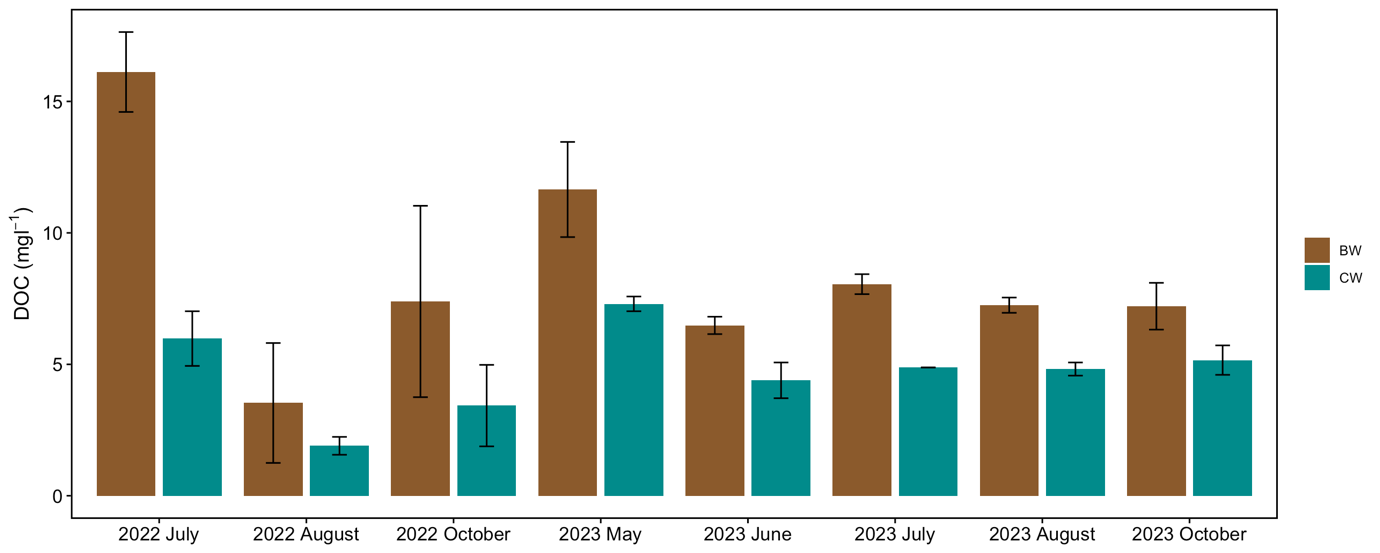


**Figure S2.** DOC concentration of water samples. Standard error shown by error bars.


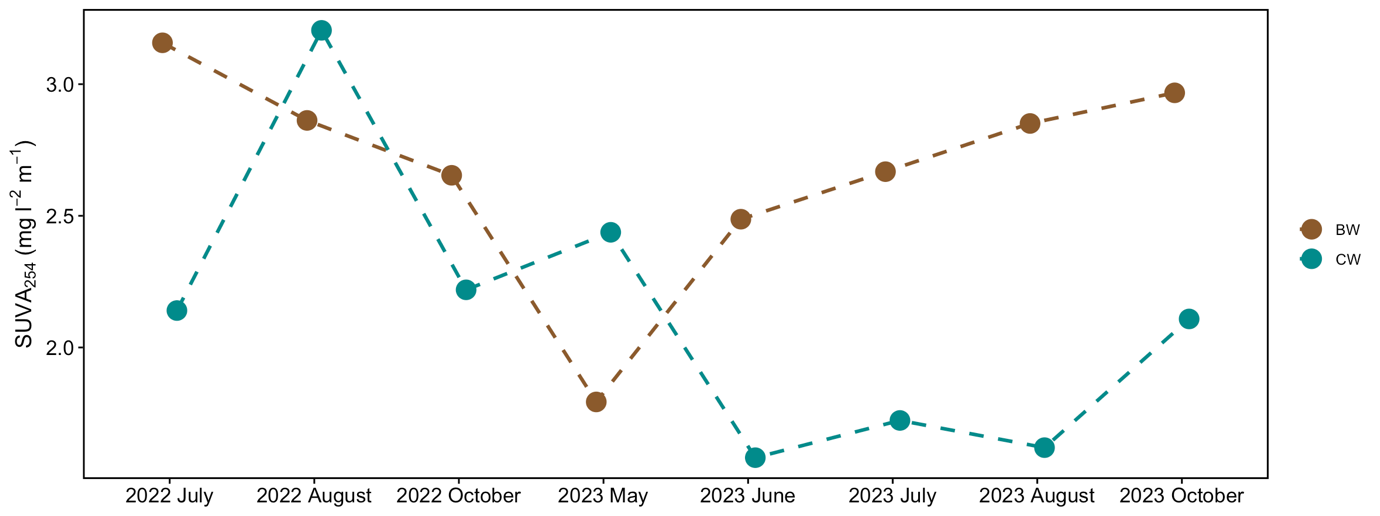


**Figure S3.** Suva_254_ concentration throughout the sampling campaign. Brown colour represents brown water river (BW), and blue represents clear water river (CW).

**
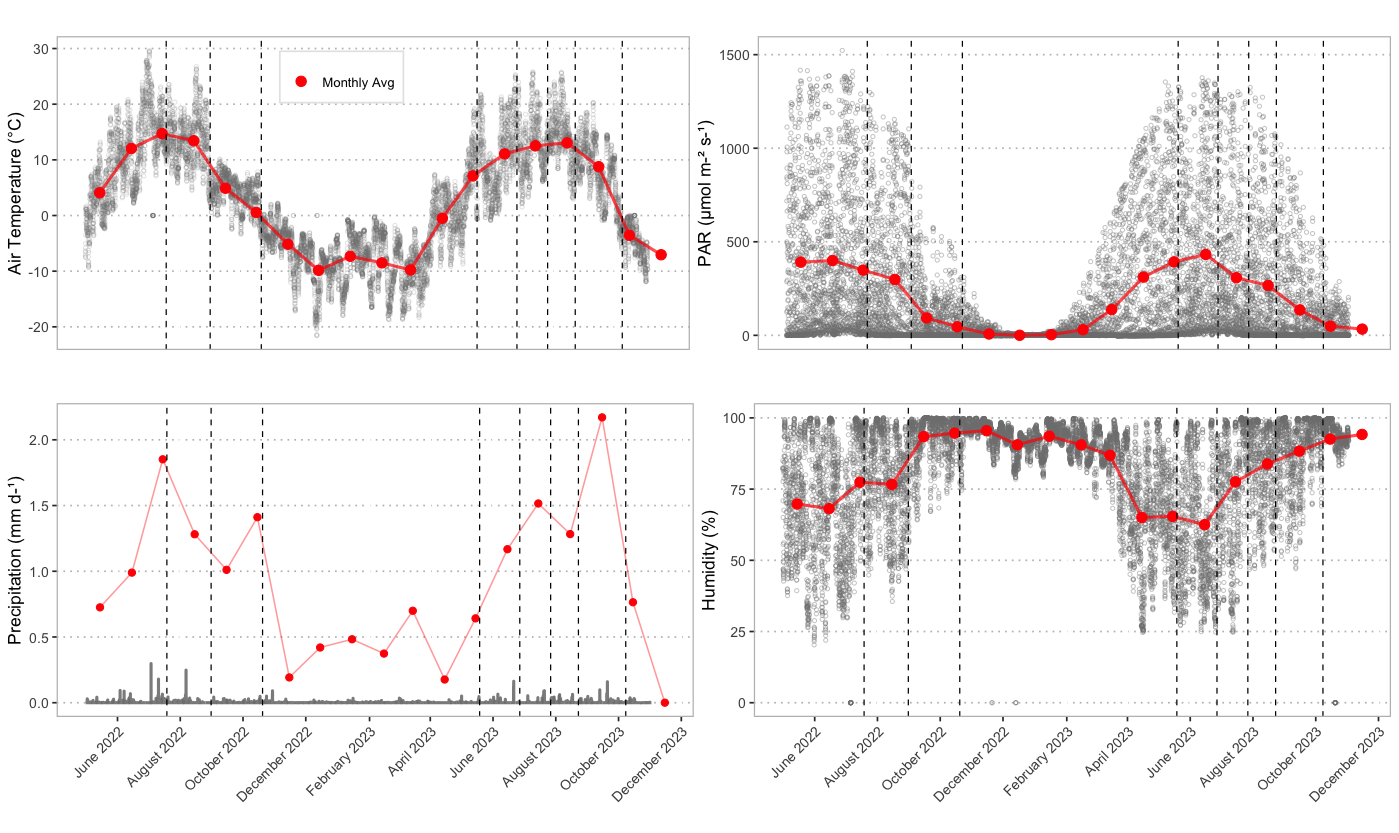
**

**Figure S4**. Meteorological data for the site from April 2022 to December 2023. Air temperature (°C), precipitation (mm d^-1^), PAR (µmol m^-2^ s^-1^), and humidity (%). Red dots show the monthly average value. Vertical dashed lines indicate measurement campaigns.


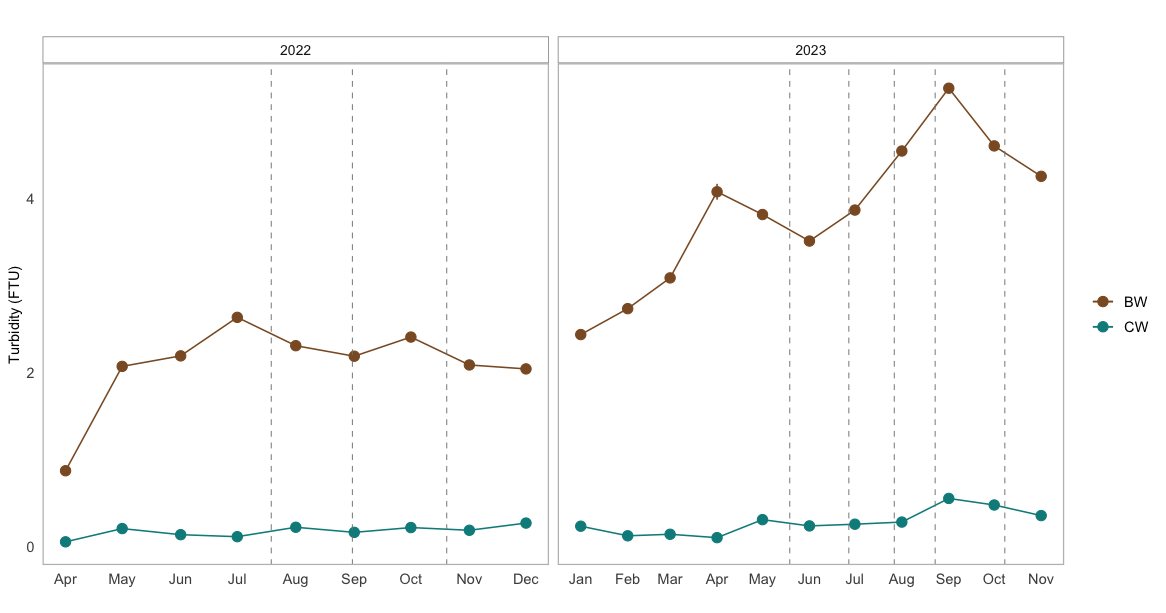


**Figure S5**. Turbidity continuous data from both rivers. Vertical dashed lines indicate measurement campaigns.


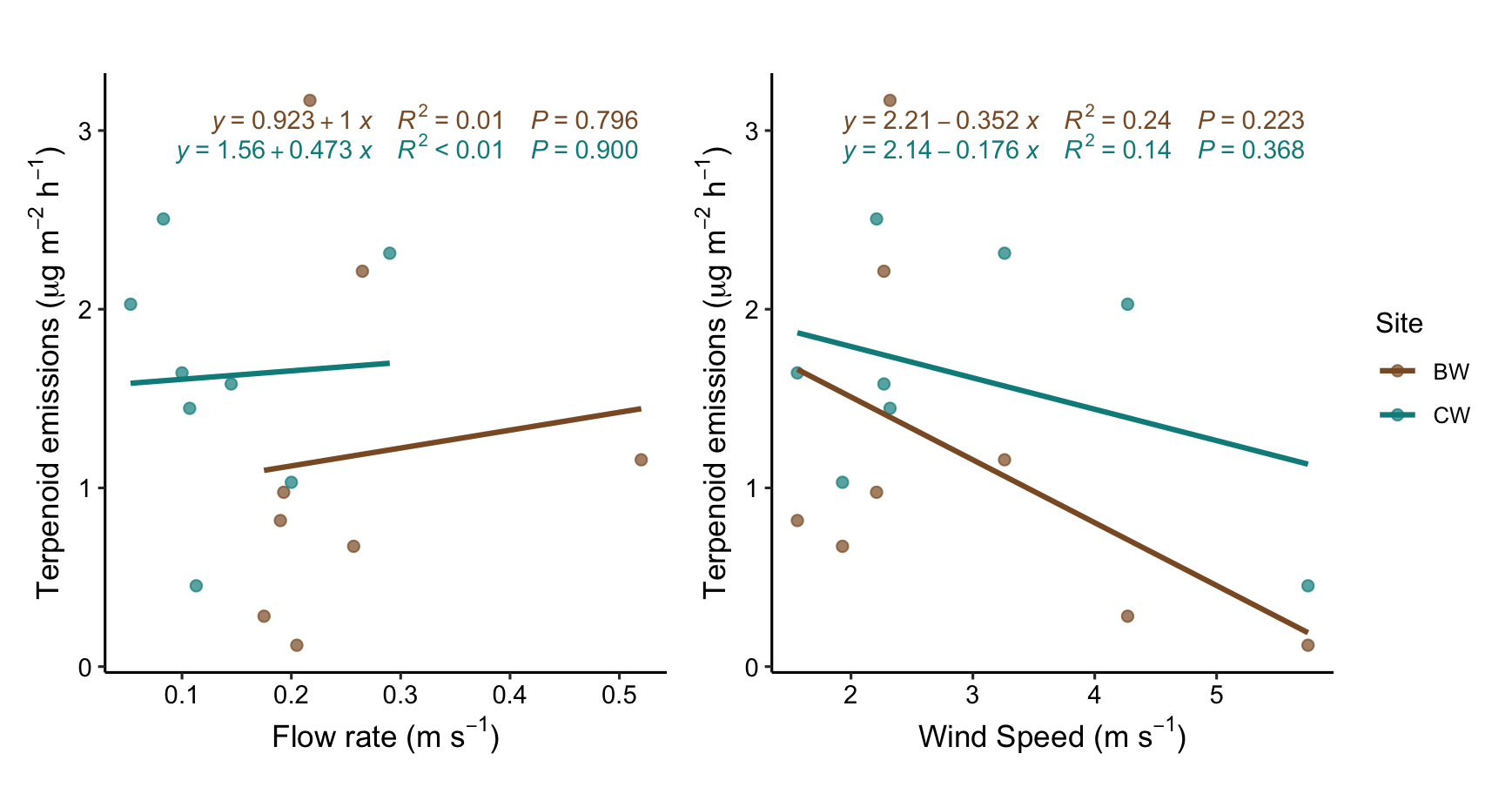


**Figure S6**. Regression line of terpenoid emission with flow rate (left) and wind speed (right).

**Table S2.** Final linear regression model (1-10) of the monoterpene emissions, examining the relationship between environmental predictors and MT emissions. Statistics include R-squared (R2), adjusted R-squared (Adj.R2), overall model F-statistic with p-value, Akaike Information Criterion (AIC), and Bayesian Information Criterion (BIC) and variables.

**Table S3.** Final linear regression model (1-7) of the sesquiterpene emissions, examining the relationship between environmental predictors and SQT emissions. Statistics include R-squared (r.squared), adjusted R-squared (adj.r.squared), sigma (residual standard error (σ)), overall model F-statistic with p-value, Akaike Information Criterion (AIC), and Bayesian Information Criterion (BIC).

**Table S4.** Post hoc pairwise comparisons of month and years using Tukey's Honestly Significant Difference (HSD) test. The table shows the mean difference, 95% confidence interval, and adjusted p-value for each pair of groups. Significant differences (p<0.05) are noted.
